# *ENHO*, *RXRA*, and *LXRA* polymorphisms and dyslipidaemia, related comorbidities and survival in haemodialysis patients

**DOI:** 10.1186/s12881-018-0708-4

**Published:** 2018-11-09

**Authors:** Alicja E. Grzegorzewska, Leszek Niepolski, Monika K. Świderska, Adrianna Mostowska, Ireneusz Stolarek, Wojciech Warchoł, Marek Figlerowicz, Paweł P. Jagodziński

**Affiliations:** 1 Department of Nephrology, Transplantology and Internal Diseases, Poznan University of Medical Sciences (PUMS), Poznań, Poland; 2 Department of Physiology, PUMS, Poznań, Poland; 3 Department of Biochemistry and Molecular Biology, PUMS, Poznań, Poland; 4 Polish Academy of Sciences, Institute of Bioorganic Chemistry, Poznań, Poland; 5 Department of Ophthalmology and Optometry, PUMS, Poznań, Poland

**Keywords:** Adropin, Dyslipidaemia, Epistatic interactions, *ENHO*, Haemodialysis, *LXRA*, *RXRA*, Survival, Transcription factor binding sites

## Abstract

**Background:**

The energy homeostasis-associated gene (*ENHO*), retinoid X receptor alpha gene (*RXRA*), and liver X receptor alpha gene (*LXRA*) are involved in adipogenic/lipogenic regulation. We investigated whether single-nucleotide polymorphisms in these genes (*ENHO* rs2281997, rs72735260; *RXRA* rs749759, rs10776909, rs10881578; *LXRA* rs2279238, rs7120118, rs11039155) are associated with dyslipidaemia, related comorbidities and survival of haemodialysis (HD) patients also tested for T-helper (Th) cell interleukin genes (*IL*).

**Methods:**

The study was carried out in 873 HD patients. Dyslipidaemia was diagnosed by the recommendations of the Kidney Disease Outcomes Quality Initiative (K/DOQI) guidelines (2003); atherogenic dyslipidaemia was referred to if the TG/HDL cholesterol ratio was equal to or higher than 3.8. Genotyping of *ENHO* SNPs, *LXRA* SNPs, and *IL12A* rs568408 was carried out using HRM analysis. *RXRA* SNPs, *IL12B* rs3212227, and *IL18* rs360719 were genotyped using PCR-RFLP analysis. The circulating adropin concentration was determined in 126 patients by enzyme-linked immunosorbent assay. Survival probability was analysed using the Kaplan-Meier method in 440 patients followed through 7.5 years.

**Results:**

Dyslipidaemia by K/DOQI was diagnosed in 459 patients (91% revealed hyper-LDL- cholesterolaemia), atherogenic dyslipidaemia was diagnosed in 454 patients, and 231 patients were free of dyslipidaemia by both criteria. The variant allele (T) of *ENHO* rs2281997 was associated with the hyper-LDL cholesterolaemic pattern of dyslipidaemia by K/DOQI. The frequency of atherogenic dyslipidaemia was lower in T-allele bearers than in CC-genotype patients. The rs2281997 T allele was associated with lower cardiovascular mortality in HD patients showing atherogenic dyslipidaemia. *ENHO, RXRA,* and *LXRA* showed epistatic interactions in dyslipidaemia. Circulating adropin was lower in atherogenic dyslipidaemia than in non-atherogenic conditions. *RXRA* rs10776909 was associated with myocardial infarction. Bearers of *LXRA* rs2279238, rs7120118 or rs11039155 minor alleles showed higher mortality. *ENHO* SNP positions fell within the same DNase 1 hypersensitivity site expressed in the Th1 cell line. Epistatic interactions occurred between rs2281997 and Th1 *IL* SNPs (rs360719, rs568408).

**Conclusions:**

Atherogenic dyslipidaemia occurs in HD patients in whom *ENHO* encodes less adropin. *ENHO*, *RXRA*, and *LXRA* SNPs, separately or jointly, are associated with dyslipidaemia, myocardial infarction, and survival in HD patients. Differences in the availability of transcription binding sites may contribute to these associations.

**Electronic supplementary material:**

The online version of this article (10.1186/s12881-018-0708-4) contains supplementary material, which is available to authorized users.

## Background

Dyslipidaemias are manifested by the elevation of total cholesterol (TC), low-density lipoprotein (LDL)-cholesterol and triglyceride (TG) concentrations, and a decrease in high-density lipoprotein (HDL)-cholesterol concentration in the blood. In 1990, atherogenic dyslipidaemia was defined as a serum lipid profile comprising the higher proportion of small dense LDL particles (LDL phenotype B), reduced HDL cholesterol, and increased TG [1]. The TG/HDL cholesterol ratio ≥ 3.8 (also referred to as the atherogenic index) was found to be reliable to identify LDL phenotype B in men and women [2] and became a marker of atherogenic dyslipidaemia closely associated with the measures of adiposity [3], cardio-metabolic risk [4], and even mortality risk [5].

Hypertriglyceridaemia and reduced HDL cholesterol plasma levels are the most commonly observed lipid abnormalities among end-stage renal disease (ESRD) patients, including haemodialysis (HD) subjects. Hypertriglyceridaemia is primarily a consequence of delayed lipid catabolism [6] due to the decreased lipoprotein lipase activity because of the downregulation of the enzyme gene [7] and presence of lipase inhibitors [8]. In HD patients, elevated hepatic production of TG-rich lipoproteins due to increased insulin resistance [6, 9] and the use of low-molecular heparins for anticoagulation [10] may also play a contributory role in hypertriglyceridaemia. A reduced HDL cholesterol level can be attributed to the acquired deficiency of lecithin-cholesterol acyltransferase, which facilitates the esterification of free cholesterol in HDL particles [11] and increased activity of cholesteryl ester transfer protein [12], which is responsible for the transport of cholesterol esters from HDL to TG-rich lipoproteins. The plasma levels of total and LDL cholesterol are generally normal among HD patients; however, the proportion of small dense LDL particles is increased [13]. According to the National Kidney Foundation/Kidney Disease Outcomes Quality Initiative (K/DOQI) clinical practice guidelines for managing dyslipidaemias in chronic kidney disease, dyslipidaemia referred to a serum LDL cholesterol level ≥ 100 mg/dL or simultaneously occurring non-HDL cholesterol ≥130 mg/dL and TG ≥ 200 mg/dL [14]. The TG/HDL cholesterol ratio is also used in HD patients as an indicator of atherogenic dyslipidaemia, and its values in the highest quintiles were prognostic for mortality in the incident and prevalent HD patients [15, 16].

Studies on the genetic background of dyslipidaemia add much to understand the prevalence and pathophysiological aspects of lipid abnormalities. The expression of hepatic lipogenic genes and adipose tissue peroxisome proliferator-activated receptor (PPAR) gamma is involved in the regulation of lipogenesis [17]. PPARs are members of the nuclear hormone receptor superfamily that act as ligand-inducible transcription factors by interacting with the peroxisome proliferator response element on the promoter of target genes. PPARs heterodimerize with retinoid X receptors (RXRs) to regulate target gene activation. The heterodimerization of PPARs with RXRs is a prerequisite for their DNA binding activity and target gene activation [18]. RXR alpha (RXRα) and liver X receptor alpha (LXRα), a nuclear receptor involved in cholesterol and TG metabolism [19], form a functional heterodimer LXRα-RXRα in which RXRα is the active ligand-binding subunit [20]. Stimulation of LXRα suppresses the hepatic expression of the energy homeostasis-associated gene (*ENHO*), a protein-coding gene located in chromosome 9p13.3. A hormone encoded by *ENHO* is adropin, discovered in 2008 [17]. *ENHO* expression in peripheral blood mononuclear cells was negatively correlated with the TG levels in Behçet’s disease [21]. In HD patients, major homozygotes of *ENHO* rs2281997 (bearers of two C alleles) were suggested to have higher circulating adropin [22].

The data mentioned above [17–20] suggest that *ENHO*, RXRα gene (*RXRA*), and LXRα gene (*LXRA*) are associated individually or jointly with lipid metabolism. Genetic variations could modulate the contribution of these genes to lipid homeostasis. Therefore, also in HD patients, *ENHO*, *RXRA*, and *LXRA* single-nucleotide polymorphisms (SNPs) might be connected directly or indirectly with dyslipidaemia, comorbidities that frequently occur together with lipid abnormalities (coronary artery disease - CAD, myocardial infarction, end-stage diabetic nephropathy), and even survival probability. In this study, for genotyping, we have chosen *ENHO* rs2281997 and rs72735260, *RXRA* rs749759, rs10776909 and rs10881578, and *LXRA* rs2279238, rs7120118 and rs11039155 SNPs. The evidence concerning the chosen SNPs in relation to dyslipidaemia and associated comorbidities is scarce and does not concern the HD population [23–25].

Adropin is required for metabolic homeostasis and is involved in preventing dyslipidaemia [17]. In human subjects, circulating adropin was demonstrated to be negatively correlated with the levels of plasma TG [26, 27], free fatty acids, apolipoprotein B, and LDL cholesterol and positively correlated with the HDL cholesterol level [26]. The serum adropin level was found to be correlated negatively with CAD [28]. Circulating adropin was found to be lower in dyslipidaemic HD patients than in non-dyslipidaemic subjects [22]. Adropin, the *ENHO* protein product, which was demonstrated to directly associate with serum lipids, was also determined in the current study to further understand the associations of *ENHO* SNPs with lipid abnormalities.

In silico methods to predict transcription factor (TF) binding sites (TFBS) are powerful approaches to detect functional SNPs. Their identification may lead to better understanding of the molecular mechanisms underlying the pathogenesis of tested abnormalities and suggest possible associations between genes—e.g., T-helper (Th) interleukin genes (*IL*)—and their protein products—e.g., interleukins (ILs). Alterations of the TFBS by SNPs can potentially affect downstream processes controlled by TF binding and can provide links to biological processes related to these TFs [29].

Th cells produce ILs that are mainly involved in immunoregulation. However, murine Th clones express transcripts for PPAR gamma 1 isoforms, and ligands for PPAR gamma mediate the inhibition of IL-2 secretion involved in Th0 polarisation to Th1 [30]. Thus, interactions between the Th clones and lipids may also occur in humans. The *IL18* rs2043055 TT genotype was associated with higher serum TG levels after an oral test in young men with fathers with an early myocardial infarction history [31]. *IL18* encodes IL-18, a potent Th1 cytokine. Compared with *IL18*(+/+) mice producing IL-18, *IL18* (−/−) mice developed hypercholesterolaemia, hyper-HDL cholesterolaemia, and hypertriglyceridaemia [32]. For clinical practice, these findings may contribute to the explanation of the connections between lipid abnormalities and inflammation that are frequently observed in atherosclerosis [33].

Assuming that *ENHO*, *RXRA*, and *LXRA* SNPs may be involved in dyslipidaemia, related comorbidities or the mortality of HD patients, we planned the genotyping of *ENHO* rs2281997 and rs72735260, *RXRA* rs749759, rs10776909 and rs10881578, and *LXRA* rs2279238, rs7120118 and rs11039155 SNPs and determined the circulating adropin concentration in HD patients to show their relevance in the lipid-related pathology of ESRD requiring dialysis treatment. In the case of significant associations among *ENHO*, *RXRA*, and *LXRA* SNPs, we aimed to perform the in silico prediction of TFBS overlapping the examined SNPs to show their potential regulatory impacts through modification of the TFBS motifs. Moreover, in a case of TFBS identification, we planned to perform gene-gene interaction analysis between *ENHO*, *RXRA*, or *LXRA* and genes possibly associated with them by sharing the same TFBS, if such genes were previously genotyped in the tested subjects.

## Patients and methods

### Patients

Prevalent HD patients (*n* = 950) who underwent dialysis at 22 dialysis centres in the Greater Poland region of Poland were evaluated as candidates for this cross-sectional study. However, when secondary causes of dyslipidaemia (hypothyroidism, alcohol abuse, medication with anticonvulsants, corticosteroid therapy) and cachectic conditions causing decreases in serum lipids (neoplasms, enteropathies, liver cirrhosis) were applied as the exclusion criteria, 77 patients were excluded. Patients had to be in a stable general condition for at least one month before enrolment. Finally, the study group consisted of 873 HD patients. The data for this study were collected from January 2009 to May 2015.

The study group consisted of 873 patients; 418 (47.9%) were treated with low-flux HD, 412 (47.2%) with high-flux HD, and 43 (4.9%) with *on-line* haemodiafiltration. Equilibrated Kt/V was maintained in all patients between 1.1 and 1.3.

The principal dietary and pharmacological treatment of all patients was based on a standard of care according to the physician.

Patients treated with antilipaemic medication were not excluded from the study (the exception: subjects included in a prospective study, see below) if they had available serum lipid profiles prior to the commencement of antilipaemic therapy that could be used as a characteristic for these patients. Subjects treated with antilipaemic agents prior to the study enrolment, in whom such a treatment was discontinued during renal replacement therapy **(**RRT), were included in the study if they did not receive antilipaemic agents at least for 6 months prior to enrolment. Since November 2013, when the guidelines of the Kidney Disease: Improving Global Outcomes (KDIGO) Work Group [34] were published, antilipaemic medication was not generally initiated in HD patients if they were not receiving it at the time of dialysis initiation.

In all HD patients, therapeutic efforts were aimed at reaching the normal serum concentrations of calcium and phosphorus. To achieve these targets, patients received phosphate binders (calcium carbonate or calcium acetate, occasionally sevelamer hydrochloride). Among vitamin D supplements, alfacalcidol was the most frequently used. Cinacalcet hydrochloride was administered in patients with serum parathyroid hormone levels equal to or exceeding 500 pg/ml. Parathyroidectomy (PTX) was performed if possible (no clinical contraindications, written informed consent obtained).

Patients also used erythropoietin-stimulating agents, iron supplements, and water-soluble vitamins. Comorbid conditions were treated as needed.

A prospective study was conducted in 440 HD patients. They were selected from 532 patients without a history of renal transplantation who started the prospective study in January 2009 [35, 36]. The initial data of the HD patients included in the prospective study were also incorporated into the cross-sectional study. Patients selected for the present study did not receive lipid-lowering medication and were tested for *ENHO*, *RXRA*, and *LXRA* SNPs. Subjects from the entire group of 873 persons who underwent genotyping for *RXRA* SNPs had been previously included in the group being retrospectively evaluated in relation to survival [37]. These patients also underwent genotyping for SNPs in Th-cell cytokine-associated genes, including Th-cell *IL*s associated with Th1 cells: *IL12A* rs568408, *IL12B* rs3212227, and *IL18* rs360719.

Patients were examined for the evidence of dyslipidaemia according to the K/DOQI guidelines [14]. Patients diagnosed as dyslipidaemic by serum LDL cholesterol ≥100 mg/dL were referred to as hyper-LDL cholesterolaemic, whereas those showing non-HDL cholesterol ≥130 mg/dL and TG ≥200 mg/dL are described as hyper-TG/hyper-non-HDL cholesterolaemic. The remaining patients are referred to as non-dyslipidaemic by K/DOQI criteria.

To approach the atherogenic pattern of dyslipidaemia, we used the TG/HDL cholesterol ratio (the atherogenic index). A ratio ≥ 3.8 was considered to indicate atherogenic dyslipidaemia [2]. HD subjects with a TG/HDL cholesterol ratio < 3.8 are described as patients without atherogenic dyslipidaemia.

Patients taking antilipaemic treatment were considered to have the type of dyslipidaemia diagnosed prior to the initiation of antilipaemic medication using the mentioned criteria.

All the study subjects were Caucasians.

The patient outcome (death, renal transplantation, moving to a non-collaborating centre) was evaluated in July 2016.

### Laboratory examinations

In all HD patients, fasting blood samples were collected before the midweek dialysis session for genotyping, serum lipid levels (TC, HDL cholesterol, TG), which were measured using enzymatic colorimetric tests (Roche Diagnostics, Mannheim, Germany), and laboratory parameters routine for HD patients. In 126 non-smoking patients dialyzed with low-flux HD, the circulating adropin levels were determined by enzyme-linked immunosorbent assay (Cusabio, Wuhan, China). HD patients tested for adropin were not receiving antilipaemic medications.

The LDL cholesterol concentration was calculated using the Friedewald formula. In patients with serum TG concentrations ≥400 mg/dL, LDL cholesterol was measured directly (BioSystems S.A., Reagents and Instruments, Barcelona, Spain). Non-HDL cholesterol was the TC minus HDL cholesterol.

### Genotyping

SNPs in *ENHO*, *RXRA*, and *LXRA* were identified from public databases such as the NCBI dbSNP database (http://www.ncbi.nlm.nih.gov/ projects/SNP/) and 1000 Genomes Browser (http://browser.1000 genomes.org/ index.html). The selection of SNPs was based on functional significance (with a preference for SNPs located in putative regulatory regions), association with metabolic disturbances in previous studies, observed linkage disequilibrium (LD) patterns and minor allele frequency (MAF) over 5% in the Caucasian population.

The characteristics of the analysed SNPs are shown in Additional file 1: Table S1. Genomic DNA was isolated in a blinded fashion from blood lymphocytes using the salt-out extraction procedure. Genotyping of *ENHO* SNPs (rs2281997, rs72735260) and *LXRA* SNPs (rs2279238, rs7120118, rs11039155) was carried out using high-resolution melting curve (HRM) analysis using the Light Cycler 480 system (Roche Diagnostics, Mannheim, Germany). Briefly, DNA fragments amplified using specific primers were subjected to HRM with 0.1 °C increments at temperatures ranging from 70 to 92 °C. *RXRA* SNPs were genotyped using polymerase chain reaction (PCR)-restriction fragment length polymorphism (RFLP) analysis as previously described [37]. The conditions of HRM and RFLP analyses are shown in Additional file 1: Table S2.

For quality control, approximately 20% of the randomly chosen samples were re-genotyped. Samples with ambiguous results were excluded from further statistical analyses. The sample size was lowest for rs72735260 (*n* = 848).

Additionally, after obtaining the results of in silico prediction revealing that *ENHO* SNP positions fell within the same DNase 1 hypersensitivity site expressed in the Th1 cell line, we included in the study previously tested Th1 cell cytokine gene SNPs (*IL12A* rs568408, *IL12B* rs3212227, and *IL18* rs360719) to check gene-gene interactions. Genotyping of *IL12A* rs568408 was performed using HRM analysis; *IL12B* rs3212227 and *IL18* rs360719 were genotyped by PCR-RFLP analysis, as previously described [37] (Additional file 1: Table S2).

The tested polymorphisms were distributed in concordance with Hardy-Weinberg equilibrium (HWE).

### In silico TFBS prediction

The potential regulatory impacts of the tested SNPs through modifications of TFBS motifs were assessed using ENCODE TFBS ChIP-seq data [38] and in silico prediction of DNA-binding sites collected in HOCOMOCO version 9 [39], JASPAR CORE version 5.0 ALPHA 2016 [40] and CIS-BP version 1.02 [41] databases with FIMO software version 4.11.1 [42].

We performed computational analysis on the GenBank DNA sequences (contigs NT_008470.20, NT_008413.19 and NT_009237.19) [43] adjacent to the SNP positions as in our previous study [44] with a *p*-value < 0.0005 and a q-value < 0.05 selected as cut-off values for reliable predictions. The motifs matched in both orientations were also analysed with respect to perfect reverse complement formation, thus increasing the true-positive match probability. Details of in silico analysis are presented in Additional file 1: Supplementary Methods.

### Statistical analysis

The results are presented as percentages for categorical variables or medians and ranges for continuous variables that were non-normally distributed by the Shapiro–Wilk test.

To compare continuous variables, the Mann–Whitney U test was used. Dichotomous variables were compared using Chi^2^ test, Chi^2^ test with Yates correction, Chi^2^ test for trend in proportions, and Fisher’s exact test, as appropriate. HWE was analysed using Chi-squared test. The occurrence of selected phenotypes with respect to the tested polymorphisms was compared using models of inheritance (dominant, recessive, and additive).

The Spearman’s rank test was used to show correlations between selected variables.

Survival probability was analysed with respect to the tested polymorphisms in patients enrolled in the prospective study [35, 36] using the Kaplan-Meier method with the log-rank test. All analyses were performed using patient groups separated by genotypes and three models of inheritance. The Cox proportional hazards model was applied to show whether and to which extent the effect of a unit change in a covariate was multiplicative with respect to the hazard rate (HR) of death. HRs were adjusted for clinical data using Cox proportional hazards regression analysis. Gender, age, RRT duration prior to the beginning of the prospective study, CAD, diabetic nephropathy, and body mass index (BMI) were applied as clinical variables possibly contributing to survival probability.

Logistic regression was used to determine the associations of selected SNPs with appropriate phenotypes among other patient characteristics (gender, age, RRT duration, CAD, diabetic nephropathy, and BMI). Only in the case of adropin, one variable (BMI) was used for adjustment due to the smaller number of analysed subjects, especially if subgroups categorized by lipidaemic status were evaluated.

A value of *P* < 0.05 was considered significant for HWE, the log-rank test, the Cox model, and logistic regression. In comparisons among demographic, clinical, and laboratory data, noncorrected *P*-values are shown. In evaluations of genetic associations, differences significant at a P-value < 0.05 were corrected using Bonferroni correction based on the critical P-value of 0.05 divided by the number of statistical tests being performed in each set of data separately to avoid missing significant associations among multiple analyses. If a *P*-value for the tested difference was equal to or lower than that shown using Bonferroni correction, the tested difference was considered statistically significant. Bonferroni correction values were approximated to the first significant number and are shown in footnotes to tables, as appropriate. Only *P* values significant after Bonferroni correction were further analysed unless otherwise stated.

The abovementioned statistical analyses were performed using Graph-Pad InStat 3.10, 32 bit for Windows (GraphPad Software, Inc., San Diego, California, United States) and Statistica version 12 (Stat Soft, Inc., Tulsa, Oklahoma, United States).

The power to detect the genetic associations was determined using Quanto v.1.2.4 software [45].

Haplotype frequencies were estimated using Haploview 4.2 software (http://www.broad.mit.edu/mpg/haploview/). Epistatic interactions between the tested SNPs were analysed using the multifactor dimensionality reduction (MDR) method [46]. Statistical significance in both tests was assessed using the 1000-fold permutation test.

Due to complex human genetic associations, in which many genes may be associated with the phenotype to some extent, we additionally evaluated the reproducibility of genetic associations for candidate loci using the Better Associations for Disease and GEnes (BADGE) system [47] and compared the results using the Bonferroni corrected *P*-value of 0.0004 obtained for 8 tested SNPs, 5 phenotypes (2 types of dyslipidaemia, CAD, myocardial infarction, diabetic nephropathy), and 3 models of inheritance.

## Results

### Patient characteristics

According to the K/DOQI criteria, 459 dyslipidaemic patients (52.6% of the total HD group) were enrolled**.** Atherogenic dyslipidaemia was diagnosed in 454 patients (52.0% of the total group)**.** The demographic, clinical and laboratory data of HD patients stratified by dyslipidaemia using K/DOQI guidelines or the atherogenic index are shown in Table 1. The combined data of all patients are presented in Additional file 1: Table S3.

**Table 1:** Demographic, clinical and laboratory data of HD patients stratified by type of dyslipidaemia

Parameter	Dyslipidaemia	Without dyslipidaemia	P value^a^	Atherogenic dyslipidaemia	Without atherogenic dyslipidaemia	
	by K/DOQI criteria			TG/HDL cholesterol ratio		*P* value^a^
				5.98 (3.81–30.83)	2.52 (0.47–3.79)	
Demographic data	*N* = 459	*N* = 414		*N* = 454	*N* = 419	
Male gender, *n*, % of all	242 (52.7)	247 (59.7)	0.039*	263 (57.9)	226 (53.9)	0.235
Age, years	66.8 (17.9–95.9)	67.1 (17.1–92.3)	0.944	66.3 (17.1–92.3)	68.3 (21.5–95.9)	0.162
Clinical data						
Main cause of ESRD						
Diabetic nephropathy, *n*, % of all	125 (27.2)	123 (29.7)	0.418	132 (29.1)	116 (27.7)	0.649
Hypertensive nephropathy, *n*, % of all	95 (20.7)	83 (20.0)	0.878	97 (21.4)	81 (19.3)	0.509
Chronic glomerulonephritis, *n*, % of all	66 (14.4)	68 (16.4)	0.457	65 (14.3)	69 (16.5)	0.431
Chronic tubulointerstitial nephritis, *n*, % of all	41 (8.9)	41 (9.9)	0.708	45 (9.9)	37 (8.8)	0.666
Coronary artery disease, n, % of all	160 (34.9)	160 (38.6)	0.246	188 (41.4)	132 (31.5)	0.002*
RRT duration, years	5.5 (0.2–28.3)	5.9 (0.1–27.5)	0.143	5.8 (0.1–28.1)	5.6 (0.2–28.3)	0.444
HD re-started after graft loss, n, % of all	25 (5.4)	22 (5.3)	0.970	25 (5.5)	22 (5.3)	0.778
Life with graft, years	8.7 (0.0–21.0)	2.1 (0.0–17.0)	0.014*	8.3 (0.0–21.0)	3.0 (0.0–20.0)	0.082
Dialysis duration, years	5.4 (0.2–28.3)	5.9 (0.1–27.3)	0.009*	5.7 (0.1–27.3)	5.6 (0.2–28.3)	0.613
LF-HD, n, % of all	224 (48.8)	194 (46.9)	0.566	199 (43.8)	219 (52.3)	0.013*
Chronic hepatitis B, n, % of all	5 (1.1)	8 (1.9)	0.555	6 (1.3)	7 (1.7)	0.884
Chronic hepatitis C, n, % of all	31 (6.8)	22 (5.3)	0.455	29 (6.4)	24 (5.7)	0.790
BMI, kg/m^2^	25.6 (12.8–59.2)	24.6 (14.3–63.5)	0.030*	26.4 (14.3–59.2)	24.2 (12.8–63.5)	< 0.000001*
BMI > 30 kg/m^2^ (obesity), n, % of all	91 (22.8)	51 (15.5)	0.016*	102 (26.1)	40 (11.8)	< 0.00001*
Laboratory data						
HDL cholesterol, mg/dL	40 (16–103)	39.1 (6–103)	0.719	33 (6–82)	47 (19–103)	< 0.000001*
Triglycerides, mg/dL	172.5 (40.6–856)	121 (29.8–585)	< 0.00001*	201 (46–856)	109 (29.8–260)	< 0.000001*
LDL cholesterol, mg/dL	120.1 (41.8–512)	73.4 (13.3–99.5)	< 0.00001*	102.4 (17–512)	93 (13.3–255)	0.0004*
Non-HDL cholesterol, mg/dL	158.7 (53.6–593)	99 (27–157)	< 0.00001*	148 (44–593)	116.7 (27–282)	< 0.000001*
Adropin, ng/mL^b^	2.02 (0.22–8.01)	2.15 (0.76–8.46)	0.491	1.85 (0.22–6.98)	2.46 (0.87–8.46)	0.002*
ALT, IU/L	14 (0.6–195)	13 (2–131)	0.243	13 (2–195)	14 (0.6–135)	0.922
PTH, pg/mL	407.7 (7.3–3757)	355.3 (13.7–2991.5)	0.082	413 (19.5–3118.3)	361.4 (7.3–3757)	0.063

^a^The Mann-Whitney U test was used to compare continuous variables. Chi-squared test with Yates correction was applied to compare dichotomous variables

^b^Determined in 126 patients: 67 with dyslipidaemia and 59 without dyslipidaemia by K/DOQI criteria; 73 with atherogenic dyslipidaemia and 53 without atherogenic dyslipidaemia

Abbreviations: *ALT* alanine aminotransferase, *BMI* body mass index, *ESRD* end-stage renal disease, *LF-HD* low flux haemodialysis, *HD* haemodialysis, *PTH* parathyroid hormone, *RRT* renal replacement therapy, *TC* total cholesterol, *TG* triglycerides

*P*-values below 0.05 are indicated by an asterisk

Gender, age, RRT duration, the prevalence of diabetic nephropathy, hypertensive nephropathy, CAD, and myocardial infarction were analysed concerning all tested SNPs. The results are shown in the supplementary material (Additional file 1: Tables S4 and S5 for *ENHO* SNPs; Additional file 1: Table S6 – S8 for *RXRA* SNPs; Additional file 1: Table S9 – S11 for *LXRA* SNPs). The chosen patient characteristics revealed multiple associations (Additional file 1: Table S12).

During the prospective follow-up, 280 patients died, 52 underwent renal transplantation, and 4 moved to non-collaborating centres. In adjusted analyses, CAD (*P* = 0.003) and diabetic nephropathy (*P* = 0.017) were associated with all-cause mortality of HD patients. Neither dyslipidaemia by K/DOQI nor atherogenic dyslipidaemia was associated with all-cause mortality (Additional file 1: Table S13).

### *ENHO* rs2281997 and tested phenotypes

The variant (T) allele of *ENHO* rs2281997 was associated with LDL cholesterol levels ≥100 mg/dL (Table 2 and Additional file 1: Table S14). There were no correlations of this allele with non-HDL cholesterol concentrations ≥130 mg/dL (Additional file 1: Table S15) and TG ≥ 200 mg/dL (Additional file Additional file 1: Table S16). The T allele of rs2281997 was associated with dyslipidaemia by K/DOQI (Additional file 1: Table S17), namely, with the hyper-LDL cholesterolaemic pattern of this dyslipidaemia (Table 2, Additional file 1: Table S18), but not with other components of dyslipidaemia by K/DOQI (Additional file 1: Tables S19 and S20). The TG/HDL cholesterol ratio was lower in T-allele bearers than in CC genotype patients (3.5, 0.5–25.5 vs. 3.9, 0.6–30.8; *P* = 0.031), and atherogenic dyslipidaemia was inversely associated with the T allele (Table 2, Additional file 1: Table S21).

**Table 2:** Significant associations between the tested polymorphisms and analysed phenotypes

Gene	SNP	Phenotypes	Model of inheritance or MAF^a^	OR (95% CI)	*P*-value	Sample power (%)	BADGE class for genetic association
*RXRA*	rs749759	MI vs without MI	Recessive	2.22 (1.28–3.84)	0.004	75.6	Fifth
*RXRA*	rs10776909	MI vs without MI	Recessive	3.08 (1.60–5.93)	0.0004	85.1	Fourth
			Additive	3.17 (1.63–6.18)	0.0004	99.9	Fourth
*ENHO*	rs2281997	Dyslipidaemia vs non-dyslipidaemia by K/DOQI criteria	Recessive	2.87 (1.65–4.99)	0.0001	99.9	Third/Fourth
			Additive	3.16 (1.80–5.57)	0.00003	99.9	Third
			MAF (T vs. C allele)	1.49 (1.21–1.84)	0.0002		Fourth
*ENHO*	rs2281997	Hyper-LDL cholesterolaemic dyslipidaemia vs non-dyslipidaemia by K/DOQI criteria	Dominant	1.55 (1.14–2.11)	0.006	78.3	Fifth
			Recessive	3.10 (1.71–5.63)	0.0001	86.5	Third/Fourth
			Additive	3.53 (1.91–6.53)	0.00003	99.9	Third
			MAF (T vs. C allele)	1.59 (1.25–2.02)	0.0002		Fourth
*ENHO*	rs2281997	LDL cholesterol ≥100 mg/dL vs LDL cholesterol < 100	Dominant	1.43 (1.10–1.87)	0.009	74.3	Fifth
			Recessive	2.18 (1.32–3.60)	0.002	75.0	Fifth/Fourth
			Additive	2.44 (1.45–4.10)	0.0006	99.9	Fourth
			MAF (T vs. C allele)	1.43 (1.16–1.76)	0.0007		Fourth
*ENHO*	rs2281997	Atherogenic dyslipidaemia vs without atherogenic dyslipidaemia	dominant	0.65 (0.50–0.85)	0.002	88.7	Fifth/Fourth
			additive	0.49 (0.29–0.81)	0.005	99.9	Fifth
			MAF (T vs. C allele)	0.70 (0.57–0.86)	0.0007		Fourth

Abbreviations: *MAF* minor allele frequency, *MI* myocardial infarction

^a^ MAF is referred to the frequency of the respective major allele. The minor allele is underlined

Detailed analyses for all tested associations are shown in the Additional file 1

Possessors of the TT genotype of *ENHO* rs2281997 showed higher LDL cholesterol concentrations than the remaining patients (111, 36–180 mg/dL vs. 97, 13.3–512 mg/dL; *P* = 0.008) but a simultaneously lower TG/HDL cholesterol ratio (3.0, 0.7–10.9 vs. 3.8, 0.5–30.8 mg/dL; *P* = 0.001) due to a higher HDL cholesterol concentration (44.5, 24–73 mg/dL vs. 39, 6–103 mg/dL; *P* = 0.0004). Patients with the TT genotype showed an approximately 3-fold higher risk of dyslipidaemia by K/DOQI in the recessive and additive models of inheritance (Table 2 and Additional file 1: Table S17).

Parameters chosen as explanatory variables for dyslipidaemia by K/DOQI included gender, age, RRT duration, diabetic nephropathy, BMI, and the TT genotype of *ENHO* rs2281997. The significant variables associated with this type of dyslipidaemia were the TT genotype of rs2281997 (OR: 2.94, 95% CI: 1.60–5.38, *P* = 0.0005) and male gender (OR: 0.72, 95% CI: 0.54–0.98, *P* = 0.035). Hyper-LDL cholesterolaemic dyslipidaemia was associated with the TT genotype of rs2281997 (OR: 2.94, 95% CI: 1.65–5.23, *P* = 0.0002) and was inversely associated with diabetic nephropathy (OR: 0.56, 95% CI: 0.38–0.83, *P* = 0.004).

Patients with atherogenic dyslipidaemia showed a higher frequency of the CC genotype than patients without this type of dyslipidaemia (56.2% vs. 45.6%, respectively; *P* = 0.002; Additional file 1: Table S21).

The parameters chosen as explanatory variables for atherogenic dyslipidaemia included gender, age, RRT duration, diabetic nephropathy, BMI, and the CC genotype of *ENHO* rs2281997. The variables associated with atherogenic dyslipidaemia were the CC genotype of rs2281997 (OR: 1.52, 95% CI: 1.13–2.06, *P* = 0.006) and BMI (OR: 1.09, 95% CI: 1.05–1.12, *P* = 0.000001).

Selected patient data, including CAD, myocardial infarction, and end-stage diabetic nephropathy, did not differ concerning *ENHO* rs2281997 polymorphisms (Additional file 1: Table S4).

**Fig. 1:**
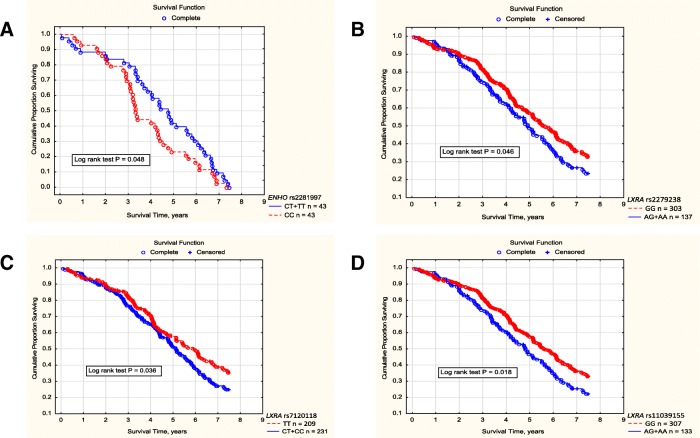
Cumulative proportion surviving of HD patients with respect to selected *ENHO* and *LXRA* SNPs. **a** Cardiovascular mortality among HD patients showing atherogenic dyslipidemia with respect to *ENHO* rs2281997 polymorphic variants (the dominant model of inheritance). Cardiovascular mortality was analyzed among 227 patients who started the prospective study showing atherogenic dyslipidemia. Through 7.5 years of follow-up, 86 patients died due to cardiovascular complications (43 subjects possessed the CC genotype and 43 patients were harboring the T allele of ENHO rs2281997). Cardiovascular mortality was analyzed using the Kaplan-Meier method with the subsequent log-rank test. In the Cox analysis, the T allele was associated with approximately 1.6-fold lower cardiovascular mortality in HD patients with atherogenic dyslipidemia (HR 0.64, 95% CI 0.42 - 0.99, *P* = 0.047). **b** Survival probability in HD patients with respect to *LXRA* rs2279238 polymorphic variants (the dominant model of inheritance). Survival probability was analyzed using the Kaplan-Meier method in 440 patients prospectively followed through 7.5 years. In the Cox analysis, HR was 1.30, 95% CI 1.01 - 1.67, *P* = 0.042. **c** Survival probability in HD patients with respect to *LXRA* rs7120118 polymorphic variants (the dominant model of inheritance). Survival probability was analyzed using the Kaplan-Meier method in 440 patients prospectively followed through 7.5 years. In the Cox analysis, HR was 1.29, 95% CI 1.02 - 1.65, *P* = 0.037. **d** Survival probability in HD patients with respect to *LXRA* rs11039155 polymorphic variants (the dominant model of inheritance). Survival probability was analyzed using the Kaplan-Meier method in 440 patients prospectively followed through 7.5 years. In the Cox analysis, HR was 1.36, 95% CI 1.05 - 1.75, *P* = 0.016

*ENHO* rs2281997 genotypes (also analysed in the models of inheritance) were not associated with all-cause or cardiovascular mortality (Additional file 1: Table S22). However, among patients with atherogenic dyslipidaemia, cardiovascular mortality was lower in patients with the TT genotype of *ENHO* rs2281997 than in those with the CC genotype (log rank *P* = 0.011), lower in patients with the TT versus CT + CC genotype (log rank *P* = 0.046), and lower in those with the CT + TT versus CC genotype (log rank *P* = 0.048) (Fig. 1a). Survival of T allele bearers in the 7.5-year prospective study was 4.76, 0.07–7.44 years, while the CC genotype patients lived 3.33, 0.62–7.33 years (*P* = 0.048). In multivariate analysis, a positive predictor of cardiovascular survival in atherogenic patients was the T allele of *ENHO* rs2281997 (HR: 0.52, 95% CI: 0.32–0.86, *P* = 0.011), whereas BMI (HR: 1.09, 95% CI: 1.03–1.16, *P* = 0.005) and CAD (HR: 1.97, 95% CI: 1.16–3.34, *P* = 0.012) indicated a worse cardiovascular survival.

### *ENHO* rs72735260 and tested phenotypes

*ENHO* rs72735260 variants showed no direct association with the type of dyslipidaemia (Additional file 1: Tables S17 and S21), analysed comorbidities (Additional file 1: Table S5), and mortality of HD patients (Additional file 1: Table S22).

### *ENHO* haplotypes and tested phenotypes

Significant associations were found only with dyslipidaemia (Table 3). The *ENHO* rs72735260_rs2281997 GT haplotype compared with rs72735260_rs2281997 GC haplotype and all other haplotypes pooled together was associated with an approximately 1.5-fold higher frequency of dyslipidaemia by K/DOQI. The *ENHO* rs72735260_rs2281997 GC haplotype was associated with atherogenic dyslipidaemia.

**Table 3:** Haplotypes of the tested genes concerning the analysed phenotypes in HD patients

Gene	Polymorphisms	Haplotype	Freq.	Case, Control Frequencies	Chi Square	P Value	P_corr_ Value^a^	OR (95% CI), p value^b^	OR (95% CI), p value^c^
myocardial infarction = CASES, without myocardial infarction = CONTROLS									
*LXRA*	rs2279238_rs7120118	GT	0.693	0.646, 0.705	4.810	0.028	0.078	reference	0.761 (0.596–0.971), 0.028
		AC	0.168	0.165, 0.169	0.036	0.849	0.993	1.068 (0.777–1.468), 0.685	0.973 (0.713–1.328), 0.863
		GC	0.139	0.190, 0.126	9.791	0.002	0.005*	1.645 (1.202–2.252), 0.002	1.624 (1.194–2.208), 0.002*
	rs11039155_rs2279238_rs7120118	GGT	0.692	0.646, 0.704	4.565	0.033	0.098	reference	0.771 (0.602–0.989), 0.040
		AAC	0.160	0.157, 0.160	0.033	0.857	1.000	1.064 (0.769–1.472), 0.709	0.976 (0.710–1.342), 0.883
		GGC	0.137	0.184, 0.124	8.828	0.003	0.005*	1.598 (1.162–2.198), 0.004	1.580 (1.156–2.159), 0.004*
dyslipidaemia by K/DOQI criteria = CASES, without dyslipidaemia by K/DOQI criteria = CONTROLS									
*ENHO*	rs72735260_rs2281997	GC	0.582	0.549, 0.619	8.786	0.003	0.008*	reference	0.756 (0.624–0.917), 0.004*
		GT	0.278	0.314, 0.238	12.299	5.0E-4	0.001*	1.483 (1.191–1.846), 0.0004	1.471 (1.189–1.819), 0.0004*
		TC	0.133	0.128, 0.138	0.434	0.5101	0.773	1.045 (0.785–1.390), 0.7645	0.921 (0.698–1.215), 0.562
atherogenic dyslipidaemia = CASES, without atherogenic dyslipidaemia = CONTROLS									
*ENHO*	rs72735260_rs2281997	GC	0.582	0.618, 0.544	9.824	0.002	0.004*	reference	1.343 (1.109–1.627), 0.003*
		GT	0.278	0.244, 0.314	10.532	0.001	0.004*	0.687 (0.553–0.854), 0.0007	0.704 (0.570–0.869), 0.001*
		TC	0.133	0.132, 0.133	0.002	0.968	1.000	0.880 (0.661–1.172), 0.3825	0.994 (0.753–1.312), 0.966
*LXRA*	rs11039155_rs2279238	GG	0.827	0.803, 0.853	7.496	0.0062	0.009*	reference	0.714 (0.550–0.927), 0.011*
		AA	0.161	0.182, 0.137	6.433	0.0112	0.028*	1.401 (1.079–1.819), 0.0111	1.401 (1.079–1.819), 0.011*
	rs2279238_rs7120118	GT	0.693	0.676, 0.711	2.423	0.1196	0.249	reference	0.846 (0.689–1.039), 0.110
		AC	0.168	0.190, 0.145	6.363	0.0117	0.029*	1.386 (1.069–1.797), 0.0135	1.391 (1.077–1.796), 0.011*
		GC	0.139	0.134, 0.145	0.424	0.5151	0.795	0.978 (0.741–1.291), 0.8776	0.819 (0.699–1.207), 0.541
	rs11039155_rs2279238_rs7120118	GGT	0.692	0.676, 0.708	2.094	0.1479	0.416	reference	0.873 (0.709–1.074), 0.199
		AAC	0.160	0.180, 0.137	5.942	0.0148	0.023*	1.369 (1.049–1.785), 0.0204	1.384 (1.065–1.798), 0.015*
		GGC	0.137	0.129, 0.145	0.917	0.3382	0.750	0.935 (0.706–1.236), 0.6351	0.882 (0.669–1.162), 0.370

Significant *P*-values are indicated using an asterisk

^a^The *p*-value was calculated using the permutation test and 1000 permutations

^b^The most common haplotype was used as the reference

^c^All other haplotypes pooled together were used as the reference

Only *ENHO* and *LXRA* haplotypes yielded significant results after correction

### Adropin, the *ENHO* protein product

In the entire group of HD patients tested for adropin, significant correlations were found between the circulating adropin levels and TG level (*r* = − 0.302, *P* = 0.0006), TG/HDL cholesterol ratio (*r* = − 0.301, P = 0.0006), and BMI (*r* = − 0.316, *P* = 0.0004). HD subjects with the CC genotype of *ENHO* rs2281997 showed higher circulating adropin levels than the remaining patients (2.38, 0.22–8.46 ng/mL vs 1.84, 0.64–5.99 ng/mL, respectively; uncorrected *P* = 0.020; P adjusted for BMI = 0.006; dominant model of inheritance; Additional file 1: Figure S1).

In patients dyslipidaemic by K/DOQI, the circulating adropin level was higher in subjects with the *ENHO* rs2281997 CC genotype than in T-allele bearers (2.55, 0.22–8.01 ng/mL vs. 1.72, 0.64–3.96 ng/mL, respectively; uncorrected *P* = 0.031; P adjusted for BMI = 0.016; Additional file 1: Figure S1). These concentrations were not significant compared with those in patients free of dyslipidaemia with the CC genotype (2.75, 0.93–8.46 ng/mL; *n* = 15; uncorrected *P* = 0.308; P adjusted for BMI = 0.300) or bearing the T allele of rs2281997 (2.23, 1.09–5.99 ng/mL; uncorrected *P* = 0.128; P adjusted for BMI = 0.286).

In patients with atherogenic dyslipidaemia, there was no difference in circulating adropin between subjects with the CC genotype and those bearing the T allele (2.11, 0.22–6.98 ng/mL vs. 1.70, 0.64–4.89 ng/mL; *P* = 0.145; P adjusted for BMI = 0.052; Additional file 1: Figure S1). The atherogenic patients with the CC genotype showed a lower adropin level than those with the CC genotype subjects free of dyslipidaemia (uncorrected *P* = 0.041; Additional file 1: Figure S1). Similarly, bearers of the T allele with atherogenic dyslipidaemia showed a lower adropin level than subjects free of dyslipidaemia harbouring the T allele (uncorrected *P* = 0.044; Additional file 1: Figure S1). However, the latter two significant *P*-values became not significant after adjustment for BMI (*P* = 0.057 for the CC genotype; *P* = 0.239 for the T allele; Additional file 1: Figure S1).

### *RXRA* rs749759 and tested phenotypes

The *RXRA* rs749759 variants were associated neither with dyslipidaemia by K/DOQI criteria (Additional file 1: Table S23) nor with atherogenic dyslipidaemia (Additional file 1: Table S24). An association was found between *RXRA* rs749759 and myocardial infarction using the BADGE system (Table 2); however, it became not significant after Bonferroni correction (Additional file 1: Table S25). Other clinical variables did not correlate with rs749759 variants (Additional file 1: Table S6).

Patients with myocardial infarction compared with those without the condition were predominantly male, were of older age, showed a higher frequency of diabetic nephropathy, and had a greater BMI (Additional file 1: Table S26). These variables, together with RRT duration and the AA genotype of *RXRA* rs749759, were used in multivariate analysis to show independent correlation with myocardial infarction. In such a model, age (HR: 1.04, 95% CI: 1.02–1.05, *P* = 0.000003), diabetic nephropathy (HR: 2.10, 95% CI: 1.41–3.13, *P* = 0.0003), male gender (HR: 2.00, 95% CI: 1.35–2.97, *P* = 0.0005), and AA genotype (HR: 2.74, 95% CI: 1.50–5.03, *P* = 0.001) remained significant.

No association with mortality of HD patients was demonstrated for rs749759 (Additional file 1: Table S22).

### *RXRA* rs10776909 and the tested phenotypes

*RXRA* rs10776909 genotypes were not associated with dyslipidaemia by K/DOQI criteria (Additional file 1: Table S23). The *RXRA* rs10776909 genotypes were also not associated with atherogenic dyslipidaemia (Additional file 1: Table S24).

An association was found between the *RXRA* rs10776909 SNP and myocardial infarction (Table 2, Additional file 1: Tables S7 and S25). In multivariate analysis, age (OR: 1.04, 95% CI: 1.02–1.05, P = 0.000003), male gender (HR: 2.02, 95% CI: 1.36–2.99, *P* = 0.0004), diabetic nephropathy (HR: 1.97, 95% CI: 1.30–2.93, *P* = 0.0008), and TT genotype of *RXRA* rs10776909 (HR: 3.15, 95% CI: 1.49–6.68, *P* = 0.003) were associated with myocardial infarction.

*RXRA* rs10776909 SNP was not associated with the mortality of HD patients (Additional file 1: Table S22).

### *RXRA* rs10881578 and tested phenotypes

*RXRA* rs10881578 variants were not associated with dyslipidaemia by K/DOQI criteria (Additional file 1: Table S23), atherogenic dyslipidaemia (Additional file 1: Table S24), comorbidities (Additional file 1: Table S8), and the mortality of HD patients (Additional file 1: Table S22).

### *RXRA* haplotypes and tested phenotypes

Associations of *RXRA* haplotypes with the tested phenotypes, including myocardial infarction (Additional file 1: Table S27), did not reach significance.

### *LXRA* rs2279238 and tested phenotypes

*LXRA* rs2279238 variants were not associated with dyslipidaemia by K/DOQI criteria (Additional file 1: Table S28) and atherogenic dyslipidaemia (Additional file 1: Table S29). *LXRA* rs2279238 SNP did not reveal significant associations with the clinical data (Additional file 1: Table S9).

Patients bearing the minor allele of *LXRA* rs2279238 showed higher all-cause mortality than the major homozygotes of this SNP (Fig. 1b, Additional file 1: Table S22). In regression analysis, significant negative predictors of survival probability were age (HR: 1.02, 95% CI: 1.01–1.03, *P* = 0.002), CAD (HR: 1.46, 95% CI: 1.07–1.98, *P* = 0.016), and the minor allele of *LXRA* rs2279238 (HR: 1.37, 95% CI: 1.02–1.83, *P* = 0.034), while RRT duration prior to the beginning of the prospective study was a positive predictor (HR: 0.75, 95% CI: 0.70–0.80, *P* = 1.0E-17). Gender, BMI and diabetic nephropathy were not significant in this model.

### *LXRA* rs7120118 and the tested phenotypes

*LXRA* rs7120118 variants were not associated with dyslipidaemia by K/DOQI criteria (Additional file 1: Table S28), atherogenic dyslipidaemia (Additional file 1: Table S29), and clinical data (Additional file 1: Table S10).

Patients bearing the minor allele of *LXRA* rs7120118 showed higher all-cause mortality than major homozygotes (Fig. 1c, Additional file 1: Table S22). This association (HR: 1.41, 95% CI: 1.06–1.87, P = 0.016) remained significant together with age, RRT duration prior to the beginning of the prospective study, and CAD. Gender, BMI and diabetic nephropathy were not significant in this model.

### *LXRA* rs11039155 and tested phenotypes

*LXRA* rs11039155 variants were not associated with dyslipidaemia by K/DOQI criteria (Additional file 1: Table S28) and atherogenic dyslipidaemia (Additional file 1: Table S29). *LXRA* rs11039155 did not reveal significant associations with the clinical data (Additional file 1: Table S11).

Patients bearing the minor allele of rs11039155 showed higher all-cause mortality than major homozygotes (Fig. 1d, Additional file 1: Table S22). This association (HR: 1.47, 95% CI: 1.14–1.89, *P* = 0.003) was also significant together with age, RRT duration prior to the beginning of the prospective study, and CAD. Gender, BMI and diabetic nephropathy were not significant in this model.

### *LXRA* haplotypes and tested phenotypes

*LXRA* haplotypes (rs11039155_rs2279238 AA, rs2279238_rs7120118 AC, rs11039155_rs2279238_rs7120118 AAC) were associated with atherogenic dyslipidaemia (Table 3). However, only rs2279238 was associated with serum lipids. Bearers of the minor allele of rs2279238 showed higher TG concentrations in additive (177, 72–459 mg/dL vs. 141.7, 29.8–856 mg/dL, P = 0.016) and recessive (177, 72–459 mg/dL vs. 143, 29.8–856 mg/dL, *P* = 0.019) models of inheritance.

*LXRA* rs2279238_rs7120118 GC and rs11039155_rs2279238_rs7120118 GGC haplotypes were associated with a higher prevalence of myocardial infarction (Table 3). These two haplotypes comprise the C allele of rs7120118. Patients harbouring two C alleles in *LXRA* rs7120118 (minor homozygotes) showed a higher frequency of myocardial infarction than that demonstrated in the TT + CT or TT subjects; however, the difference was not significant after Bonferroni correction (*P* = 0.013 for CC vs. TT + CT and *P* = 0.011 for CC vs. TT) (Additional file 1: Table S10).

### Gene-gene interactions concerning the tested phenotypes

A gene-gene interaction was noted among the *ENHO* rs2281997, *RXRA* rs10776909, and *LXRA* rs7120118 polymorphisms in relation to dyslipidaemia by K/DOQI (Additional file 1: Table S30).

*RXRA* rs10881578 and *LXRA* rs2279238 showed gene-gene interactions concerning atherogenic dyslipidaemia (Additional file 1: Table S30).

Gene-gene interactions among the tested SNPs did not indicate significant results in relation to comorbidities, including myocardial infarction (Additional file 1: Table S31).

### In silico TFBS prediction

The ENCODE ChIP-seq dataset reported positions of *ENHO* rs72735260 and rs2281997 overlapping the same DNase 1 hypersensitivity site (DHS1) cluster expressed in the Th1 cell line. The position of *RXRA* rs10776909 was overlapped by the ENCODE transcription factor peaks for the DNA-directed RNA polymerase II subunit RPB1 (POLR2A), transcriptional repressor CTCF (CTCF), transcription factor p65 (RELA, also called p65), ETS-related transcription factor Elf-1 (Elf-1) and early B-cell factor 1 (EBF1). All ENCODE ChIP-seq peaks covering positions of the investigated SNPs and DNA binding sites of the transcription factor peaks are reported in Additional file 1: Table S32 and S33.

The analysis of TFBS prediction revealed that the minor allele of *RXRA* rs10776909 removed the TFBS of the three GR-like steroid hormone receptors—glucocorticoid receptor (NR3C1, also called GR), mineralocorticoid receptor (NR3C2, also called MR) and androgen receptor (NR3C4, also called AR)—together with TFBS for interferon regulatory factor (IRF-5) and RXR-related factors: hepatocyte nuclear factor 4-α (HNF-4-α, positive orientation) and 4-ɣ (HNF-4-ɣ, negative orientation). Additionally, for *RXRA* rs10776909, the photoreceptor-specific nuclear receptor (NR2E3, also called PNR) and myeloid zinc finger 1 (MZF-1) were reported.

The presence of the minor allele for *RXRA* rs749759 caused the removal of binding sites for several transcription factors, most importantly the specificity protein Sp-1-like factors (Sp3, Sp4).

Of the *LXRA* variants, the addition of TFBS was found for Krüppel-like factor 8 (Klf8) in rs2279238, and interferon regulatory factor 4 (IRF-4) for the rs7120118. All significantly different TFBS for the studied *ENHO*, *RXRA*, and *LXRA* variants are reported in Table 4.

**Table 4:** Results of the transcription factor binding site prediction according to the software FIMO for the tested SNPs

SNP	Allele	Transcription factor	Modification (in the presence of the minor allele)	Strand	*p*-value	q-value	Matched sequence
rs749759	G	NR0B1	Removed	“-”	1.96e-05	0.022	CCTCCCACTC
rs749759	G	Sp4	Removed	“+”	2.54e-05	0.0266	GGGGCCAGGGGAGTGgGAGGCACG
rs749759	G	ZBTB7B	Removed	“+”	6.36e-05	0.0226	GGGGCCAGGGGAGTGgGAGGCA
rs749759	G	EGR-2	Removed	“+”	8.97e-05	0.033	GGAGTGgGAGG
rs749759	G	Sp3	Removed	“-”	7.23e-05	0.0255	CCCACTCCCCT
rs72735260	T	AR	Added	“+”	2.41e-05	0.0273	AGGGAAAGAGTGtACCC
rs72735260	T	RARα::RXRα	Added	“-”	3.85e-05	0.0423	GGGTCAGGGGCCGGGTA
rs10881578	–	–	–	–	–	–	–
rs10776909	C	NR3C1	Removed	“+”	1.16e-05	0.013	GGGAcTTTGAGTTC
rs10776909	C	AR	Removed	“-”	2.96e-05	0.0333	GGGAACTCAAAGTCC
rs10776909	C	IRF-5	Removed	“-”	4.59e-05	0.0246	GAGAGGGGAACTCAAAGTCC
rs10776909	T	MZF-1	Added	“+”	6.33e-05	0.023	TGTGGGGAt
rs10776909	T	NR2E3	Added	“-”	3.34e-05	0.0364	GAACTCAAAATCCC
rs10776909	C	NR3C2	Removed	“-”	1.72e-05	0.0161	GGGAACTCAAAGTCCCC
rs10776909	C	HNF-4-α	Removed	“+”	4.13e-05	0.0455	GGGGAcTTTGAGTTC
rs10776909	C	HNF-4-ɣ	Removed	“-”	4.6e-05	0.0498	GGAACTCAAAGTCCC
rs2281997	–	–	–	“-”	–	–	–
rs2279238	A	Klf8	Added	“+”	2.29e-05	0.0263	CAGtGTGTG
rs2279238	A	ZBTB3 (*Mus musculus*)	Added	“+”	8.54e-06	0.00974	TATGCAGtG
rs7120118	C	IRF-4	Added	“-”	7.59e-06	0.00841	ACTCATGAAATGAGAAAT
rs11039155	G	ETV7	Removed	“+”	4.19e-05	0.0459	GCTCCAGgAAGAGATGT
rs11039155	G	Elf-1	Removed	“+”	4.34e-05	0.0484	GCTCCAGgAAGAG
rs11039155	G	Stat3	Removed	“+”	2.64e-05	0.0293	CTCCAGgAAG

The table contains only statistically significant in silico-predicted differentially bound transcription factors

The data on all DNA binding sites overlapping the tested SNPs identified by FIMO are presented in Additional file 1: Table S34.

### *ENHO* and Th cell cytokine genes

The positions of both tested *ENHO* SNPs overlapped the DHS1 cluster expressed in Th1 cell line. Therefore, we analysed gene-gene epistatic interactions between *ENHO* SNPs (rs2281997, rs72735260) and Th1 cell cytokine gene SNPs (*IL12A* rs568408, *IL12B* rs3212227, and *IL18* rs360719) with respect to both types of dyslipidaemia. Gene-gene interaction was shown between *ENHO* rs2281997 and *IL18* rs360719 with respect to dyslipidaemia by K/DOQI and *IL12A* rs568408 with respect to atherogenic dyslipidaemia (Additional file 1: Table S35).

*IL12A* rs568408 was associated with all-cause mortality in the 7.5-year prospective study. Bearers of the minor allele of rs568408 showed higher survival than patients showing homozygosity for the major allele (*P* = 0.028) (Additional file 1: Figure S2).

### Evaluation of the reproducibility of genetic associations

The significance of the tested associations with respect to the BADGE system [47] is shown in Table 2. According to the BADGE system [47], only third-class or fourth-class associations in combination with other evidence would indicate weak associations between a disease phenotype and alleles of the tested polymorphisms that could be retested with larger study samples. The BADGE classification indicates a significance level independent of the tested phenotype numbers. However, in this study, the Bonferroni-corrected *P* value of 0.0004 corresponded to the fourth and third BADGE classes.

## Discussion

The present study showed, that among the tested polymorphisms, the data on *ENHO* rs2281997 provided the strongest arguments for associations with serum lipids in HD patients. We have demonstrated, for the first time, that the TT genotype of *ENHO* rs2281997 was associated with dyslipidaemia by K/DOQI criteria at the third BADGE class for genetic association. Because dyslipidaemia by K/DOQI includes lipid abnormalities overlapping increased concentrations of LDL cholesterol, triglycerides, and non-HDL cholesterol [22], we examined these three components and showed that the abovementioned association depends on hyper-LDL cholesterolaemia defined as a serum LDL cholesterol concentration ≥ 100 mg/dL. The T allele (pooled CT + TT genotypes) of rs2281997 also appeared to be associated with dyslipidaemia by K/DOQI and the hypercholesterolaemic pattern of that type of dyslipidaemia but at the fourth BADGE class for genetic association. Moreover, the T allele was protective against atherogenic dyslipidaemia at the fourth BADGE class, showing a negative association with the atherogenic index.

The levels of circulating adropin, a protein product of *ENHO*, are negatively correlated with the levels of plasma LDL cholesterol [26] and TG [26, 27] and are positively with the levels of HDL cholesterol in human subjects [26]. In the examined HD patients, the levels of circulating adropin were negatively correlated with TG and the atherogenic index (the TG/HDL cholesterol ratio). However, only patients with atherogenic dyslipidaemia differed significantly in the levels of circulating adropin from the remaining patients, whereas such a difference was not observed when patients dyslipidaemic by K/DOQI were compared with the remaining patients. Although the levels of circulating adropin were associated with CAD [27, 28] and diabetic nephropathy [48] in subjects without renal failure, we did not show associations between *ENHO* SNPs and CAD, myocardial infarction, and diabetic nephropathy in HD patients.

In patients free of dyslipidaemia by both criteria, the CC genotype possessors produced more adropin than bearers of the T allele. The same coding pattern was shown in patients dyslipidaemic by K/DOQI criteria, who also did not differ with this regard from patients non-dyslipidaemic by K/DOQI showing respective polymorphic variants. Therefore, the association of *ENHO* with the hyper-LDL cholesterolaemic pattern of dyslipidaemia occurred beyond its impact on adropin production. The mechanism needs to be elucidated in further studies.

In HD patients with atherogenic dyslipidaemia, *ENHO* was significantly down-regulated in both the CC genotype and pooled CT + TT genotype patients compared with subjects without atherogenic dyslipidaemia. Among patients with atherogenic dyslipidaemia, both genotype groups (CC vs CT + TT) did not differ significantly in the levels of circulating adropin. Therefore, the down-regulation of rs2281997 expression seems to contribute to atherogenic dyslipidaemia in dialysis patients.

Patients with atherogenic dyslipidaemia showed a lower frequency of the T allele of *ENHO* rs2281997 than patients without this type of dyslipidaemia at the fourth BADGE class for genetic association. The T allele appeared protective against atherogenic dyslipidaemia, although less adropin was encoded in patients harbouring this allele. A lower atherogenic index in the TT-genotype possessors among the entire group of HD patients than that of the remaining patients was due to significantly higher HDL cholesterol levels (*P* = 0.0004) but not lower serum TG levels. Plasma TG and HDL cholesterol are included in the calculation of the atherogenic index. Serum HDL cholesterol did not correlate with circulating adropin in HD patients, but higher HDL cholesterol levels may be related to the TT genotype. Inversely, a decline in adropin function increased fasting TG in adropin-knockout mice [49], and TG was negatively correlated with adropin in HD patients. However, the serum TG concentrations were not associated with the T allele. This observation might at least partially explain the protective role of the T allele against atherogenic dyslipidaemia without the involvement of circulating adropin.

*ENHO* haplotypes were also associated with the prevalence of dyslipidaemia. These associations seem to be dependent on the T allele and C allele of rs2281997 because rs72735260 did not correlate directly with serum lipids.

A question arises whether the magnitude of adropin production is associated with *ENHO* rs2281997 genotypes in HD patients. In all HD subgroups defined by lipid status, subjects with the CC genotype of *ENHO* rs2281997 showed higher median values of circulating adropin than those harbouring the T allele, although the differences were not always statistically significant (Additional file 1: Figure S1). Small subgroup samples and an impact of confounding variables are issues for consideration. In our adropin analyses, only one variable could be hardly used for adjustment according to statistical rules. When all parameters (gender, age, RRT duration, CAD, diabetic nephropathy, and BMI) that were applied in the logistic regression analyses in our other evaluations were used in the atherogenic subgroup to compare the adropin concentration between the CC genotype and the T allele patients, the *P*-value reached significance (*P* = 0.040), indicating greater adropin production in the CC subjects also under atherogenic conditions. Therefore, the present study supports our previous suggestion that the CC rs2281997 genotype HD patients produce more adropin than those harbouring the T allele [22], although atherogenic dyslipidaemia is associated with the downregulation of adropin production.

The positions of *ENHO* rs2281997 and rs72735260 fell within the same DHS1 cluster expressed in the Th1 cell line. Analysis of DHS1 identified allele-specific interaction with multiple regulatory proteins, including nuclear respiratory factor 1, which regulates genes involved in mitochondrial and metabolic functions [50]. Epistatic interactions were shown between rs2281997 and Th1 cytokine genes: *IL18* rs360719 and *IL12A* rs568408. There is demonstrated evidence that Th1 cell cytokines such as IL-1β in type 1 diabetic patients [51], IL-18 in systemic lupus erythaematosus subjects [52] and IL-12A(p40) in overweight/obese women [53] are associated with the serum lipid profile.

Interestingly, the T allele of rs2281997 was associated with the hyper-LDL cholesterolaemic pattern of dyslipidaemia by K/DOQI criteria and simultaneously with a lower risk of atherogenic dyslipidaemia diagnosed by the atherogenic index. In HD patients, hyper-LDL cholesterolaemia was diagnosed already at LDL cholesterol concentrations equal to 100 mg/dL because these patients are at an increased risk of CAD [22]. Generally, ESRD does not influence the LDL subfraction levels [54], and survival is better in subjects with higher LDL cholesterol levels [55]. Therefore, higher LDL cholesterol levels in HD patients may not be so strongly counteractive to the lower atherogenic index in the T allele bearers. Factors attenuating dyslipidaemia such as dialysis duration (> 7 years), female gender, age (> 50 years) [56] or end-stage diabetic nephropathy in the studied patients did not abolish the predictive value of rs2281997 in hyper-LDL cholesterolaemic dyslipidaemia and atherogenic dyslipidaemia.

Concerning atherogenic dyslipidaemia, *ENHO* rs2281997 interacted with *IL12A* rs568408, which was related to all-cause mortality in the dominant mode of inheritance. *ENHO* rs2281997 did not contribute solely to all-cause or cardiovascular mortality among the entire HD group. However, in the subgroup of HD patients showing atherogenic dyslipidaemia, the T allele of *ENHO* rs2281997 was associated with a 1.6-fold lower cardiovascular mortality. Among the entire group of HD patients, this allele was associated with a hyper-LDL cholesterolaemic pattern of dyslipidaemia by K/DOQI but also with a lower atherogenic index.

The association of the T allele with hyper-LDL cholesterolaemia could be paradoxically beneficial for the survival of HD patients. In a study by Kilpatrick et al. [55], both total hypercholesterolaemia and hyper-LDL cholesterolaemia showed an association with better survival in non-black HD patients. The serum lipid profile reflects the nutritional status of HD patients [56], and inadequate nutrition may be an important contributing factor to the mortality in this group [57]. In non-dialysed heart failure subjects, better nutrition is a predictor of longer survival [58]. HD patients with higher serum cholesterol concentrations could present less pronounced protein-energy wasting, a condition associated with an increased death risk from cardiovascular diseases [59].

All HD patients of the discussed subgroup presented atherogenic dyslipidaemia, similar to those bearing the T allele of *ENHO* rs2281997. The TG/HDL cholesterol ratio (the atherogenic index) predicts cardiovascular outcomes and survival in prevalent nondiabetic dialysis patients. Patients with higher TG/HDL cholesterol levels (quintile 5) had a higher incidence of cardiovascular events, cardiovascular mortality and all-cause mortality than patients in quintile 1 [16]. In our study, CAD was significantly more frequent in the HD patients with atherogenic dyslipidaemia than in those without this type of dyslipidaemia. This phenomenon was not observed if HD patients with dyslipidaemia by K/DOQI were compared with those with non-dyslipidaemic by K/DOQI. Therefore, a lower atherogenic index in the T allele bearers indicating a less pronounced harmful type of dyslipidaemia could contribute to lower cardiovascular mortality in HD patients harbouring this allele.

All data mentioned above taken together might, at least, partially explain why the T allele of *ENHO* rs2281997, which was associated with a lower atherogenic index and hyper-LDL cholesterolaemic pattern of dyslipidaemia by K/DOQI (both reported as promoting survival in HD patients), was also associated with lower cardiovascular mortality among HD patients but only among those showing atherogenic dyslipidaemia at the beginning of our 7.5-year prospective study.

*LXRA* SNPs are not independently associated with the analysed phenotypes at the BADGE class I-IV [47]. However, *LXRA* haplotypes showed an association with the prevalence of atherogenic dyslipidaemia and myocardial infarction.

*LXRA* (also referred to *NR1H3*) encodes LXRα. LXRα upregulates hepatic lipogenic enzymes and increases blood TG levels [60]. In this study, *LXRA, ENHO*, and *RXRA* SNPs interacted in the occurrence of both types of dyslipidaemia. Stimulation of LXRα suppresses hepatic *ENHO* expression [17]; therefore, adropin production decreases what contributes to dyslipidaemia. In HD patients, lower plasma adropin levels correlate with higher TG concentrations. All three “atherogenic” *LXRA* haplotypes included the minor allele (A) of rs2279238 (p.Ser99Ser). In non-corrected analyses, bearers of the minor allele of rs2279238 showed higher TG concentrations. *LXRA* haplotypes, associated with atherogenic dyslipidaemia, were not significant concerning the prevalence of CAD or myocardial infarction, but the minor allele bearers showed higher all-cause mortality than homozygotes of the major allele. Among hypertensive Whites and Hispanics showing CAD, the minor allele of *LXRA* rs2279238 (denoted as T in this study) was associated with an increased risk of having a primary outcome (all-cause death, nonfatal myocardial infarction, or nonfatal stroke) [61]. We found that the DNA-binding site for the transcriptional factor Klf8 was added in the presence of the minor allele of rs2279238. Klf8 was associated with a poor prognosis of cancers [62].

The *LXRA* haplotypes comprising the minor allele of rs7120118 were associated with myocardial infarction, which was the strongest predictor of all-cause mortality of the prospectively analysed HD group. The IRF-4 binding site was added in the presence of the minor allele of rs7120118. However, this addition is possibly a false positive because the observation was not confirmed when the motifs were cross-analysed between databases. To our knowledge, no study has shown the association of rs11039155 with survival.

Therefore, associations of *LXRA* SNPs with survival may be explained by their correlations with atherogenic dyslipidaemia and myocardial infarction, as well as by the addition or removal of specific TFBS.

*RXRA* encodes RXRα, which is highly expressed in heart muscle. RXRα is a part of the vitamin D signalling pathway and is involved in lipid metabolic processes, cardiac muscle cell proliferation and differentiation [http://www.uniprot.org/uniprot/P19793]. In this study, *RXRA* SNPs (rs749759, rs10776909) showed an association with the prevalence of myocardial infarction but not with serum lipids. It is unknown how specific *RXRA* SNPs influence the susceptibility to myocardial infarction. Possibly, the minor homozygosity of both *RXRA* SNPs negatively influences the vitamin D signalling pathway and causes a relative (functional) vitamin D deficiency. HD patients, who usually have low vitamin D concentrations, might have been especially prone to further abnormalities in the vitamin D signalling pathway. Low plasma vitamin D concentrations are known to be associated with myocardial infarction in the general population [63]. The presence of the minor allele of rs749759 was associated with the removal of the binding site for the transcription factor Sp3. The reduced binding affinity of Sp1/Sp3 in the presence of the T allele of the tissue-type plasminogen activator –7351C > T polymorphism explained an increased risk of myocardial infarction in individuals carrying this allele [64]. Transcription factor Sp3 is suggested in our study as associated with myocardial infarction, but we cannot exclude that other TFBS, shown as related to *RXRA* SNPs, are also associated with this phenotype.

### Study limitations

Due to the financial shortage, determination of the plasma adropin concentration was performed in a limited number of subjects. Similarly, the atherogenic index, instead of directly determining small, dense LDL cholesterol particles, was used as an approach of atherogenic dyslipidaemia.

The phenotypes analysed in our study obviously depended not only on tested polymorphisms but are influenced by multiple confounding variables. Although uraemic state ameliorates many specific signs and symptoms of diseases leading to HD-dependent renal failure, the heterogeneity of ESRD causes may influence the tested phenotypes. Our multivariate analyses included only diabetic nephropathy. Moreover, for smaller samples, additionally divided into subgroups, the influence of confounding factors could substantially disturb the statistical significance. Therefore, in those cases, we tried to choose patients similarly influenced by the suspected confounders. Thus, adropin was determined in non-smoking patients dialysed exclusively with low flux HD. However, residual diuresis was not collected in these patients, although circulating adropin is negatively correlated with the urine output in HD patients [22]. On the other hand, according to the medical history, the urine output in the examined patients tested for adropin was not greater than 600 mL/day compared with previously studied patients showing preserved diuresis up to 2000 mL/day. Similarly, the dialysis procedure may influence the serum components.

## Conclusions

According to the BADGE system [47], our study suggests weak associations of tested SNPs with analysed phenotypes, however, worth to be retested with larger study samples. Nevertheless, demonstrated associations were obtained with a sufficient sample power, were confirmed in multivariate analyses, corresponded with circulating adropin concentrations, and/or with results of in silico analyses. Epistatic interactions between *ENHO*, *RXRA*, and *LXRA* in both patterns of dyslipidaemia and *LXRA* haplotype analysed with respect to atherogenic dyslipidaemia are in logic concordance with previous physiological studies [17, 19, 20]. Therefore, we conclude that our findings indicate that *ENHO*, *RXRA*, and *LXRA* are involved in the genetic architecture of dyslipidaemia in HD patients. Associations between *ENHO* and dyslipidaemia, *RXRA* and myocardial infarction as well as *LXRA* and survival of HD patients might be the inspiration for further detailed investigations of these relationships. Exploring the *ENHO*-adropin axis in atherogenic dyslipidaemia may result in findings leading to conclusions important for treatment of dyslipidaemia and prevention of its consequences.

## Additional file

Additional file 1: Detailed methods and results. (DOCX 367 kb)

## Abbreviations

ALT: Alanine aminotransferase
BADGE: Better Associations for Disease and Genes
CAD: Coronary artery disease
CTCF: Transcriptional repressor CTCF
DHS1: DNase 1 hypersensitivity site cluster
EBF1: Early B-cell factor 1
Elf-1: ETS-related transcription factor Elf-1
*ENHO*: Energy homeostasis-associated gene
HD: Haemodialysis
HDL: High-density lipoprotein
HNF-4-ɣ: Hepatocyte nuclear factor 4- ɣ
HNF-4-α: Hepatocyte nuclear factor 4-α
HR: Hazard rate
HRM: High-resolution melting curve
HWE: Hardy-Weinberg equilibrium
IL: Interleukin
IRF-4: Interferon regulatory factor 4
IRF-5: Interferon regulatory factor 5
K/DOQI: Kidney Disease Outcomes Quality Initiative
Klf8: Krüppel-like factor 8
LDL: Low-density lipoprotein
LF-HD: Low flux haemodialysis
LXRα: Liver X receptor alpha
MAF: Minor allele frequency
MDR: Multifactor dimensionality reduction
MI: Myocardial infarction
MZF-1: Myeloid zinc finger 1
NR2E3: Also called PNR, photoreceptor-specific nuclear receptor
NR3C1: Also called GR, glucocorticoid receptor
NR3C2: Also called MR, mineralocorticoid receptor
NR3C4: Also called AR, androgen receptor
PCR: Polymerase chain reaction
POLR2A: RNA polymerase II subunit RPB1
PPAR: Peroxisome proliferator-activated receptor
PTH: Parathyroid hormone
RELA: Also called p65, transcription factor p65
RFLP: Restriction fragment length polymorphism
RRT: Renal replacement therapy
RXR: Retinoid X receptor
RXRα: Retinoid X receptor alpha
Sp: Specificity protein
TC: Total cholesterol
TFBS: Transcription factor binding sites
TG: Triglyceride
Th: T helper
