# Supplementary material for: ENHO, RXRA, and LXRA polymorphisms and dyslipidaemia, related comorbidities and survival in haemodialysis patients
Source: BMC Med Genet. 2018 Nov 9;19:194. doi: 10.1186/s12881-018-0708-4 (PMC6234788; doi:10.1186/s12881-018-0708-4)
Supplement: Supplementary file 1 — Detailed methods and results. (DOCX 367 kb) [file 12881_2018_708_MOESM1_ESM.docx]

**Additional files**

The contents page

[Additional file 1: Supplementary Methods 4](#_Toc528457191)

[Additional file 1: Supplementary Tables 5](#_Toc528457192)

[**Additional file 1: Table S1. Characteristics of the analyzed polymorphisms.** 5](#_Toc528457193)

[**Additional file 1: Table S2. HRM and RFLP conditions for the identification of tested polymorphisms.** 6](#_Toc528457194)

[**Additional file 1: Table S3. Demographic, clinical and laboratory data of HD patients enrolled to the study (n = 873).** 8](#_Toc528457195)

[**Additional file 1: Table S4. *ENHO* rs2281997 polymorphic variants and demographic and clinical data of HD patients (n = 873).** 10](#_Toc528457196)

[**Additional file 1: Table S5. *ENHO* rs72735260 polymorphic variants and demographic and clinical data of HD patients.** 12](#_Toc528457197)

[**Additional file 1: Table S6. *RXRA* rs749759 polymorphic variants and demographic and clinical data of HD patients (n = 868).** 14](#_Toc528457198)

[**Additional file 1: Table S7. *RXRA* rs10776909 polymorphic variants and demographic and clinical data of HD patients (n = 872).** 16](#_Toc528457199)

[**Additional file 1: Table S8. *RXRA* rs10881578 polymorphic variants and demographic and clinical data of HD patients (n = 872).** 18](#_Toc528457200)

[**Additional file 1: Table S9. *LXRA* rs2279238 polymorphic variants and demographic and clinical data of HD patients (n = 861).** 20](#_Toc528457201)

[**Additional file 1: Table S10. *LXRA* rs7120118 polymorphic variants and demographic and clinical data of HD patients (n = 860).** 22](#_Toc528457202)

[**Additional file 1: Table S11. *LXRA* rs11039155 polymorphic variants and demographic and clinical data of HD patients (n = 861).** 24](#_Toc528457203)

[**Additional file 1: Table S12. The association structure among selected phenotypes of hemodialysis patients.** 26](#_Toc528457204)

[**Additional file 1: Table S13. All-cause mortality since the start of 7.5-year prospective study in 440 HD patients stratified by selected phenotypes.** 27](#_Toc528457205)

[**Additional file 1: Table S14. Associations between *ENHO* rs2281997 polymorphic variants and LDL-cholesterol levels ≥100 mg/dL in hemodialysis patients.** 28](#_Toc528457206)

[**Additional file 1: Table S15. Associations between *ENHO* rs2281997 polymorphic variants and non-HDL-cholesterol levels ≥130 mg/dL in hemodialysis patients (n = 873).** 29](#_Toc528457207)

[**Additional file 1: Table S16. Associations between *ENHO* rs2281997 polymorphic variants and TG ≥ 200 mg/dL in hemodialysis patients (n = 873).** 30](#_Toc528457208)

[**Additional file 1: Table S17. Associations between *ENHO* rs2281997 and *ENHO* rs72735260 polymorphic variants and dyslipidemia diagnosed by K/DOQI recommendations in hemodialysis patients.** 31](#_Toc528457209)

[**Additional file 1: Table S18. Associations between *ENHO* rs2281997 polymorphic variants and hyper-LDL-cholesterolemic pattern of dyslipidemia in hemodialysis patients.** 33](#_Toc528457210)

[**Additional file 1: Table S19. Associations between *ENHO* rs2281997 polymorphic variants and simultaneously occurring hyper-TG/hyper-non-HDL-cholesterolemic** **and** **hyper-LDL-cholesterolemic patterns of dyslipidemia in hemodialysis patients.** 34](#_Toc528457211)

[**Additional file 1: S20 Table S20. Associations between *ENHO* rs2281997 polymorphic variants and hyper-TG/hyper-non-HDL-cholesterolemic** **pattern of dyslipidemia in hemodialysis patients.** 35](#_Toc528457212)

[**Additional file 1: Table S21. Distribution of *ENHO* polymorphic variants in HD patients categorized by atherogenic dyslipidemia.** 36](#_Toc528457213)

[**Additional file 1: Table S22. Mortality of HD patients (n = 440) by the Kaplan-Meier analysis with P values obtained in the subsequent log-rank test for models of inheritance as well as multiple P values obtained in comparisons of mortality in patients separated by genotypes**. 38](#_Toc528457214)

[**Additional file 1: Table S23. Associations between *RXRA* polymorphic variants and dyslipidemia diagnosed by K/DOQI recommendations in hemodialysis patients.** 39](#_Toc528457215)

[**Additional file 1: Table S24. Associations between *RXRA* polymorphic variants and atherogenic dyslipidemia in hemodialysis patients.** 41](#_Toc528457216)

[**Additional file 1: Table S25. Associations between *RXRA* polymorphic variants and myocardial infarction (MI) in HD patients.** 43](#_Toc528457217)

[**Additional file 1: Table S26. Demographic, clinical and laboratory data of hemodialysis patients with myocardial infarction (MI) and without myocardial infarction (non-MI).** 45](#_Toc528457218)

[**Additional file 1: Table S27. Distribution of *RXRA* haplotypes in HD patients with myocardial infarction (cases) and HD patients without myocardial infarction (controls).** 46](#_Toc528457219)

[**Additional file 1: Table S28. Associations between *LXRA* polymorphic variants and dyslipidemia diagnosed by K/DOQI recommendations^2^ in hemodialysis patients.** 47](#_Toc528457220)

[**Additional file 1: Table S29. Associations between *LXRA* polymorphic variants and atherogenic dyslipidemia in hemodialysis patients.** 50](#_Toc528457221)

[**Additional file 1: Table S30. Gene-gene interactions between tested *ENHO*, *RXRA*, and *LXRA* polymorphisms in HD patients stratified by dyslipidemia by K/DOQI criteria or atherogenic dyslipidemia.** 53](#_Toc528457222)

[**Additional file 1: Table S31. Gene-gene interactions between tested SNPs in respect of myocardial infarction occurrence.** 54](#_Toc528457223)

[**Additional file 1: Table S32. Results of the scanning of rs10881578, rs749759, rs10776909 and rs11039155 flanking sequences by FIMO software for DNA-binding sites of ENCODE ChIP-seq predicted transcription factors peaks. Table contains only statistically significant DNA-binding sites.** 55](#_Toc528457224)

[**Additional file 1: Table S33. Function annotation information for rs749759, rs72735260, rs10881578, rs10776909, rs2281997, rs2279238, rs7120118, and rs11039155 SNPs (annotation comes from dbSNP's predicted functional effect of variant on RefSeq transcripts).** 56](#_Toc528457225)

[**Additional file 1: Table S34. FIMO predicted DNA-binding sites overlapping variants positions.** 57](#_Toc528457226)

[**Additional file 1: Table S35. Gene-gene interactions between *ENHO* and T helper 1 cell cytokine gene polymorphisms by MDR analysis.** 61](#_Toc528457227)

[Additional file 1: Supplementary Figures 62](#_Toc528457228)

[**Additional file 1: Figure S1 Adropin concentrations according to types of dyslipidemia and *ENHO* rs2281997 genotypes. 63**](#_Toc528457229)

[**Additional file 1: Figure S2. Survival probability in HD patients in respect of *IL12A* rs568408 polymorphic variants (the dominant model of inheritance)** 64](#_Toc528457233)

# Additional file 1: Supplementary Methods

**FIMO input files**

Direct sequences containing one of the alleles were selected from GenBank contig NT_008470.20 for the rs749759, rs10881578, rs10776909, contig NT_008413.19 for the rs72735260 and rs2281997 and from contig NT_009237.19 for the rs2279238, rs7120118 and rs11039155. Sequences were flanking 150 bp up- and downstream of the variant’s position. When searching for binding sites of ChIP-seq predicted transcription factors flanking sequences were broaden +- 700 bp.

**Background files**

Background files were calculated directly from input FASTA sequences with MEME suit fasta-get-markov script. The following, are the model letter frequencies used with FIMO background files when scanning for differential TFBS.

rs10881578: A 0.185 C 0.315 G 0.315 T 0.185

rs72735260: A 0.202 C 0.298 G 0.298 T 0.202

rs749759: A 0.184 C 0.316 G 0.316 T 0.184

rs2281997: A 0.200 C 0.300 G 0.300 T 0.200

rs10776909: A 0.175 C 0.325 G 0.325 T 0.175

rs2279238: A 0.208 C 0.291 G 0.291 T 0.208

rs7120118: A 0.280 C 0.220 G 0.220 T 0.280

rs11039155: A 0.235 C 0.265 G 0.265 T 0.235

To improve sensitivity for the binding sites detection of the ChIP-seq annotated transcription factor peaks we applied default random letter frequencies model for the background files (A 0.275 C 0.225 G 0.225 T 0.275).

# Additional file 1: Supplementary Tables

## **Additional file 1: Table S1. Characteristics of the analyzed polymorphisms.**

| Gene symbol | rs no. | Location^a^ | | SNP function^b^ | | Alleles^c^ | | MAF^d^ | | MAF^e^ | |
| --- | --- | --- | --- | --- | --- | --- | --- | --- | --- | --- | --- |
| *ENHO* | rs2281997 | chr9:34521867 | | Intron | | C / T | | 0.353 | | 0.354 | |
| *ENHO* | rs72735260 | chr9:34521188 | | UTR-3 | | G / T | | 0.098 | | NA | |
| *RXRA* | rs10881578 | chr9:137232535 | | Intron | | A / G | | 0.289 | | 0.239 | |
| *RXRA* | rs10776909 | chr9:137288746 | | Intron | | C / T | | 0.215 | | 0.208 | |
| *RXRA* | rs749759 | chr9:137324652 | | Intron | | A / G | | 0.234 | | 0.208 | |
| *LXRA* | rs2279238 | chr11:47282024 | | cds-synon | | A / G | | 0.152 | | 0.146 | |
| *LXRA* | rs7120118 | chr11:47286290 | | Intron | | C / T | | 0.297 | | 0.301 | |
| *LXRA* | rs11039155 | chr11:47280762 | | UTR-5 | | A / G | | 0.143 | | NA | |
| *IL12A* | rs568408 | chr3:159713467 | | UTR-3 | | A / G | | 0.117 | | NA | |
| *IL12B* | rs3212227 | chr5:158742950 | | UTR-3 | | A / C | | 0.223 | | 0.190 | |
| *IL18* | rs360719 | chr11:112036149 | | Near Gene-5 | | A / G | | 0.278 | | NA | |
| a - NCBI build 37 / hg19. | |  |  | |  | |  | |  | |  |
| b - According to the Single Nucleotide Polymorphism database (dbSNP). | | | | | | |  | |  | |  |
| c - Underline denotes the minor allele. | |  |  | |  | |  | |  | |  |
| d - MAF, minor allele frequecy (1000 Genomes project, EUR samples) | | | | |  | |  | |  | |  |
| e - MAF, minor allele frequency (HapMap, CEU samples) | | | | |  | |  | |  | |  |

## **Additional file 1: Table S2. HRM and RFLP conditions for the identification of tested polymorphisms.**

|  |  |  |  |  |  | HRM analysis | RFLP analysis | | |
| --- | --- | --- | --- | --- | --- | --- | --- | --- | --- |
| Gene symbol | rs no. | Alleles | Primers for PCR amplification  (5’ – 3’) | Annealing temp. (°C) | PCR product length (bp) | Melting temp. range (°C) | Restriction enzyme | Restriction fragment length (bp) | |
| *ENHO* | rs2281997 | C/T | F: CTCAGTGATTCCTGGGCAGT | 55.0 | 174 | 78 – 93 |  | | |
|  |  |  | R: GACCCAAGGTCTTTCACCAG |  |  |  |  | | |
| *ENHO* | rs72735260 | G/T | F: CATGGGTTCCAGTTTGCTTC | 55.0 | 174 | 82 - 97 |  | | |
|  |  |  | R: TCAGTGGCCCTAAGGAGATG |  |  |  |  | | |
| *RXRA* | rs10881578 | A/G | F: TCTTGAGCAATGCCAGCAG | 60.6 | 75 | 80 - 90 |  | | |
|  |  |  | R: CCACAGCTCACACATCCAATC |  |  |  |  | | |
| *RXRA* | rs10776909 | C/T | F: CAGCCTGTGGCCTGCTCA | 60.6 | 95 | 82 - 92 |  | | |
|  |  |  | R: AACCTCCGGCCCTTGGAG |  |  |  |  | | |
| *RXRA* | rs749759 | A/G | F: ATAGGGCTTGCCTGCCTAGA | 62.6 | 382 |  | BstXI | A = 382 | |
|  |  |  | R: CTCCACCATAGCCCAAGTGA |  |  |  |  | G = 243 + 139 | |
| *LXRA* | rs2279238 | A/G | F: ATGCTGGGGAACGACTAT | 55.0 | 76 | 70 - 90 |  | | |
|  |  |  | R: CCTCGCAGCTCAGAACATT |  |  |  |  |  |  |
| *LXRA* | rs7120118 | C/T | F: TTGGCACTTGTAGACTCATGC | 55.0 | 119 | 70 - 90 |  | | |
|  |  |  | R: TTCTCCCCAAGACCTCACTC |  |  |  |  |  |  |
| *LXRA* | rs11039155 | A/G | F: AGGACAGTGCCTTGGTAATGA | 55.0 | 55 | 73 - 89 |  | | |
|  |  |  | R: CCAGCCACAAGGACATCTCT |  |  |  |  | | |
| *IL12A* | rs568408 | A/G | F: ATGAGGAAACTTTGATAGGATG | 54.0 | 156 | 70 - 85 |  | | |
|  |  |  | R: TTCCCTTCTTAGCAATTCATTC |  |  |  |  | | |
| *IL12B* | rs3212227 | A/C | F: TTAAAGACACAACGGAATAGAC | 62.6 | 557 |  | TaqI | | A = 557 |
|  |  |  | R: TGCTTTATCAACACCATCTCC |  |  |  |  | | C = 455 + 102 |
| *IL18* | rs360719 | C/T | F: CAACAGTGATTACAAAGGAAGT | 62.6 | 474 |  | NlaIII | | T = 295 + 179 |
|  |  |  | R: TAAATGGGTAGGAATAAGTGAGA |  |  |  |  | | C = 474 |

Abbreviations: *ENHO* - energy homeostasis-associated gene, HRM analysis - High Resolution Melt analysis, *IL* – interleukin gene, *LXRA* - liver receptor X alpha gene, RFLP analysis - Restriction Fragment Length Polymorphism analysis, *RXRA* - retinoid X receptor alpha gene

## **Additional file 1: Table S3. Demographic, clinical and laboratory data of HD patients enrolled to the study (n = 873).**

| **Demographic data** |  |
| --- | --- |
| Male gender, n, % of all | 489 (56.0) |
| Age, years | 67.1 (17.1 - 95.9) |
| RRT duration, years | 5.7 (0.1 - 28.3) |
| **Cause of ESRD** |  |
| Diabetic nephropathy, n, % of all | 248 (28.4) |
| Chronic glomerulonephritis, n, % of all | 134 (15.3) |
| Hypertensive nephropathy, n, % of all | 178 (20.4) |
| Chronic tubulointerstitial nephritis, n, % of all | 82 (9.4) |
| **Clinical data** |  |
| Coronary artery disease, n, % of all | 320 (36.7) |
| Myocardial infarction, n, % of all | 185 (21.2) |
| Lipid lowering treatment, n, % of all | 22 (2.5) |
| Chronic hepatitis B, n, % of all | 13 (1.5) |
| Chronic hepatitis C, n, % of all | 53 (6.1) |
| BMI, kg/m^2^ | 25.2 (12.8 – 63.5) |
| BMI >30 kg/m^2^ (obesity) | 142 (19.5) |
| **Type of RRT** |  |
| LF-HD, n, % of all | 418 (47.9) |
| HF-HD, n, % of all | 412 (47.2) |
| HDF, n, % of all | 43 (4.9) |
| HF-HD/HDF, n, % of all | 455 (52.1) |
| PD as the first modality of RRT, n, % of all | 20 (2.3) |
| **Laboratory data** |  |
| TC, mg/dL | 174 (51 - 626) |
| HDL-cholesterol, mg/dL | 39.2 (6 - 103) |
| Triglycerides, mg/dL | 143 (29.8 - 856) |
| LDL-cholesterol, mg/dL | 98 (13.3 - 512) |
| Non-HDL-cholesterol, mg/dL | 132 (27 - 593) |
| LDL-cholesterol/HDL-cholesterol ratio | 2.5 (0.4 - 15.5) |
| HDL-cholesterol/TC ratio | 0.23 (0.05 - 0.64) |
| TG/HDL-cholesterol ratio | 3.6 (0.5 - 30.8) |
| ALT, IU/L | 13 (0.6 - 195) |
| AST, IU/L | 15 (3 - 152) |
| GGT, IU/L | 27 (1 - 682) |
| ALP, U/L | 96.5 (24 - 1684) |
| Adropin, ng/mL^a^ | 2.12 (0.22 - 8.46) |
| PTH, pg/mL | 388.8 (7.3 - 3757) |
| Ca, mg/dL | 8.9 (5.4 - 12.8) |
| P, mg/dL | 5.1 (1.8 - 11.3) |

a - determined in 126 patients

Abbreviations: ALP - alkaline phosphatase, ALT – alanine aminotransferase, AST - aspartate aminotransferase, ESRD – end-stage renal disease, GGT - gamma-glutamyl transferase, HDF - hemodiafiltration , HF-HD - high flux hemodialysis, LF-HD - low flux hemodialysis, N - number of patients, PD - peritoneal dialysis, PTH - parathyroid hormone, RRT - renal replacement therapy, TC -total cholesterol, TG - triglycerides

**Additional file 1: Table S4. *ENHO* rs2281997 polymorphic variants and demographic and clinical data of HD patients (n = 873).**

| Parameter | CC | CT | TT | Mode  of inheritance | Odds ratio (95% CI) | P value |
| --- | --- | --- | --- | --- | --- | --- |
|  | n = 446 | n = 356 | n = 71 |  |  |  |
| Male gender, n, % of all | 245 (54.9) | 211 (59.3) | 33 (46.5) | TT vs CC + CT | 0.659 (0.405, 1.072) | 0.118^b^ |
|  |  |  |  | CT + TT vs CC | 1.094 (0.837, 1.429) | 0.556^b^ |
|  |  |  |  | CC vs TT | 1.404 (0.849, 2.319) | 0.231^b^ |
| Age, years | 65.9 (17.1 - 92.3) | 67.6 (25.2 - 95.9) | 69.1 (29.7 - 88.6) | TT vs CC + CT |  | 0.135^a^ |
|  |  |  |  | CT + TT vs CC |  | 0.082^a^ |
|  |  |  |  | CC vs TT |  | 0.073^a^ |
| RRT duration, years | 5.5 (0.1 - 28) | 5.7 (0.2 - 28.3) | 6.5 (0.3 - 23) | TT vs CC + CT |  | 0.038^a,c^ |
|  |  |  |  | CT + TT vs CC |  | 0.654^a^ |
|  |  |  |  | CC vs TT |  | 0.049^a,c^ |
| Diabetic nephropathy, n, % of all | 121 (27.1) | 103 (28.9) | 24 (33.8) | TT vs CC + CT | 1.318 (0.787, 2.206) | 0.360^b^ |
|  |  |  |  | CT + TT vs CC | 1.137 (0.847, 1.526) | 0.435^b^ |
|  |  |  |  | CC vs TT | 0.729 (0.427, 1.244) | 0.308^b^ |
| Hypertensive nephropathy, n, % of all | 92 (20.6) | 69 (19.4) | 17 (23.9) | TT vs CC + CT | 1.253 (0.708, 2.220) | 0.534^b^ |
|  |  |  |  | CT + TT vs CC | 0.970 (0.698, 1.349) | 0.925^b^ |
|  |  |  |  | CC vs TT | 0.826 (0.457, 1.491) | 0.632^b^ |
| Coronary artery disease, n, % of all | 155 (34.8) | 141 (39.6) | 24 (33.8) | TT vs CC + CT | 0.873 (0.523, 1.457) | 0.695^b^ |
|  |  |  |  | CT + TT vs CC | 1.182 (0.898, 1.557) | 0.262^b^ |
|  |  |  |  | CC vs TT | 1.043 (0.615, 1.77) | 0.982^b^ |
| Myocardial infarction, n, % of all | 85 (19.1) | 88 (24.7) | 12 (16.9) | TT vs CC + CT | 0.739 (0.389, 1.407) | 0.440^b^ |
|  |  |  |  | CT + TT vs CC | 1.299 (0.938, 1.799) | 0.135^b^ |
|  |  |  |  | CC vs TT | 1.158 (0.596, 2.249) | 0.788^b^ |

a - Mann Whitney test

b - Chi - square test with Yates correction

c – Not significant after Bonferroni correction (for 1 SNP, 3 inheritance models, and 7 phenotypes, a significant Bonferroni corrected P-value is below 0.002)

**Additional file 1: Table S5. *ENHO* rs72735260 polymorphic variants and demographic and clinical data of HD patients.**

| Parameter, n = 848 | GG | GT | TT | Mode  of inheritance | Odds ratio (95% CI) | P value |
| --- | --- | --- | --- | --- | --- | --- |
|  | n = 629 | n = 201 | n = 18 |  |  |  |
| Male gender, n, % of all | 358 (56.9) | 102 (50.7) | 10 (55.6) | TT vs GG + GT | 1.005 (0.393, 2.573) | 1.000^b^ |
|  |  |  |  | GT + TT vs GG | 0.792 (0.582, 1.079) | 0.161^b^ |
|  |  |  |  | GG vs TT | 1.057 (0.412, 2.714) | 1.000^b^ |
| Age, years | 67.1 (17.9 - 92.5) | 67.1 (17.1 - 95.9) | 68.5 (28.1 - 86) | TT vs GG + GT |  | 0.975^a^ |
|  |  |  |  | GT + TT vs GG |  | 0.952^a^ |
|  |  |  |  | GG vs TT |  | 0.984^a^ |
| RRT vintage, years | 5.7 (0.1 - 28.3) | 5.7 (0.1 - 27.3) | 5.4 (0.2 - 13.1) | TT vs GG + GT |  | 0.490^a^ |
|  |  |  |  | GT + TT vs GG |  | 0.206^a^ |
|  |  |  |  | GG vs TT |  | 0.444^a^ |
| Diabetic nephropathy, n, % of all | 187 (29.7) | 50 (24.9) | 5 (27.8) | TT vs GG + GT | 0.962 (0.339, 2.729) | 1.000^b^ |
|  |  |  |  | GT + TT vs GG | 0.793 (0.559, 1.125) | 0.224^b^ |
|  |  |  |  | GG vs TT | 1.1 (0.387, 3.129) | 1.000^b^ |
| Hypertensive nephropathy, n, % of all | 129 (20.6) | 39 (19.4) | 3 (16.7) | TT vs GG + GT | 0.788 (0.226, 2.754) | 0.939^b^ |
|  |  |  |  | GT + TT vs GG | 0.92 (0.624, 1.356) | 0.745^b^ |
|  |  |  |  | GG vs TT | 1.29 (0.368, 4.523) | 0.919^b^ |
| Coronary artery disease, n, % of all | 232 (36.9) | 72 (35.8) | 10 (55.6) | TT vs GG + GT | 2.163 (0.845, 5.539) | 0.162^b^ |
|  |  |  |  | GT + TT vs GG | 1.024 (0.745, 1.408) | 0.947^b^ |
|  |  |  |  | GG vs TT | 0.468 (0.182, 1.201) | 0.172^b^ |
| Myocardial infarction, n, % of all | 132 (21) | 44 (21.9) | 5 (27.8) | TT vs GG + GT | 1.429 (0.503, 4.063) | 0.702^b^ |
|  |  |  |  | GT + TT vs GG | 1.085 (0.749, 1.573) | 0.737^b^ |
|  |  |  |  | GG vs TT | 0.691 (0.242, 1.972) | 0.687^b^ |

a - Mann Whitney test

b - Chi - square test with Yates correction

Results are presented as median and range (minimum – maximum) or number (percentage)

**Additional file 1: Table S6. RXRA rs749759 polymorphic variants and demographic and clinical data of HD patients (n = 868).**

| Parameter | GG | AG | AA | Mode  of inheritance | Odds ratio (95% CI) | P value |
| --- | --- | --- | --- | --- | --- | --- |
|  | n = 462 | n = 344 | n = 62 |  |  |  |
| Male gender, n, % of all | 258 (55.8) | 189 (54.9) | 41 (66.1) | AA vs GG + AG | 1.568 (0.910, 2.701) | 0.134^b^ |
|  |  |  |  | AG + AA vs GG | 1.033 (0.790, 1.352) | 0.865^b^ |
|  |  |  |  | AA vs GG | 0.648 (0.371, 1.131) | 0.162^b^ |
| Age, years | 68.3 (21.5 - 95.9) | 67.1 (25.2 - 95.9) | 66.8 (31.1 - 89.2) | AA vs GG + AG |  | 0.628^a^ |
|  |  |  |  | AG + AA vs GG |  | 0.493^a^ |
|  |  |  |  | AA vs GG |  | 0.540^a^ |
| RRT vintage, years | 5.6 (0.2 - 28.3) | 5.9 (0.2 - 26.9) | 5.7 (0.2 - 12.9) | AA vs GG + AG |  | 0.888^a^ |
|  |  |  |  | AG + AA vs GG |  | 0.470^a^ |
|  |  |  |  | AA vs GG |  | 0.992^a^ |
| Diabetic nephropathy, n, % of all | 145 (31.4) | 86 (25.0) | 14 (22.6) | AA vs GG + AG | 0.726 (0.393, 1.342) | 0.380^b^ |
|  |  |  |  | AG + AA vs GG | 0.714 (0.53, 0.964) | 0.033^b,c^ |
|  |  |  |  | AA vs GG | 1.568 (0.838, 2.936) | 0.204^b^ |
| Hypertensive nephropathy, n, % of all | 87 (18.8) | 77 (22.4) | 13 (21.0) | AA vs GG + AG | 1.039 (0.55, 1.96) | 1.000^b^ |
|  |  |  |  | AG + AA vs GG | 1.228 (0.882, 1.708) | 0.257^b^ |
|  |  |  |  | AA vs GG | 0.874 (0.454, 1.683) | 0.818^b^ |
| Coronary artery disease, n, % of all | 176 (38.1) | 112 (32.6) | 29 (46.8) | AA vs GG + AG | 1.581 (0.94, 2.657) | 0.109^b^ |
|  |  |  |  | AG + AA vs GG | 0.865 (0.655, 1.141) | 0.339^b^ |
|  |  |  |  | AA vs GG | 0.7 (0.411, 1.193) | 0.239^b^ |
| Myocardial infarction, n, % of all | 94 (20.3) | 66 (19.2) | 22 (35.5) | AA vs GG + AG | 2.221 (1.283, 3.842) | 0.006^b,c^ |
|  |  |  |  | AG + AA vs GG | 1.083 (0.781, 1.503) | 0.692^b^ |
|  |  |  |  | AA vs GG | 0.464 (0.263, 0.819) | 0.011^b,c^ |

a - Mann Whitney test

b - Chi - square test with Yates correction

c – non-significant after the Bonferroni correction (for 1 SNP, 3 inheritance models, and 7 phenotypes, a significant Bonferroni corrected P-value is below 0.002)

Results are presented as median and range (minimum – maximum) or number (percentage)

**Additional file 1: Table S7. RXRA rs10776909 polymorphic variants and demographic and clinical data of HD patients (n = 872).**

| Parameter | CC | CT | TT | Mode  of inheritance | Odds ratio (95% CI) | P value |
| --- | --- | --- | --- | --- | --- | --- |
|  | n = 546 | n = 287 | n = 39 |  |  |  |
| Male gender, n, % of all | 300 (54.9) | 164 (57.1) | 24 (61.5) | TT vs CC + CT | 1.272 (0.658, 2.46) | 0.581^b^ |
|  |  |  |  | CT + TT vs CC | 1.117 (0.847, 1.474) | 0.476^b^ |
|  |  |  |  | CC vs TT | 0.762 (0.391, 1.485) | 0.526^b^ |
| Age, years | 67.9 (17.1 - 93.4) | 65.6 (25.2 - 95.9) | 68.4 (31.1 - 91.9) | TT vs CC + CT |  | 0.253^a^ |
|  |  |  |  | CT + TT vs CC |  | 0.653^a^ |
|  |  |  |  | CC vs TT |  | 0.349^a^ |
| RRT vintage, years | 5.6 (0.1 - 28) | 6 (0.2 - 28.3) | 5.5 (1.4 - 13.1) | TT vs CC + CT |  | 0.541^a^ |
|  |  |  |  | CT + TT vs CC |  | 0.224^a^ |
|  |  |  |  | CC vs TT |  | 0.711^a^ |
| Diabetic nephropathy, n, % of all | 166 (30.4) | 67 (23.3) | 14 (35.9) | TT vs CC + CT | 1.442 (0.737, 2.822) | 0.372^b^ |
|  |  |  |  | CT + TT vs CC | 0.757 (0.555, 1.032) | 0.092^b^ |
|  |  |  |  | CC vs TT | 0.78 (0.396, 1.539) | 0.590^b^ |
| Hypertensive nephropathy, n, % of all | 109 (20.0) | 63 (22.0) | 6 (15.4) | TT vs CC + CT | 0.699 (0.288, 1.695) | 0.553^b^ |
|  |  |  |  | CT + TT vs CC | 1.076 (0.767, 1.51) | 0.734^b^ |
|  |  |  |  | CC vs TT | 1.372 (0.561, 3.357) | 0.627^b^ |
| Coronary artery disease, n, % of all | 208 (38.1) | 90 (31.4) | 21 (53.8) | TT vs CC + CT | 2.095 (1.099, 3.993) | 0.034^b,c^ |
|  |  |  |  | CT + TT vs CC | 0.839 (0.630, 1.118) | 0.260^b^ |
|  |  |  |  | CC vs TT | 0.527 (0.275, 1.013) | 0.076^b^ |
| Myocardial infarction, n, % of all | 107 (19.6) | 60 (20.9) | 17 (43.6) | TT vs CC + CT | 3.082 (1.600, 5.934) | 0.0009^b*^ |
|  |  |  |  | CT + TT vs CC | 1.269 (0.911, 1.768) | 0.186^b^ |
|  |  |  |  | CC vs TT | 0.315 (0.162, 0.615) | 0.0008^b*^ |

a - Mann Whitney test

b - Chi - square test with Yates correction

c – non-significant after the Bonferroni correction (for 1 SNP, 3 inheritance models, and 7 phenotypes, a significant Bonferroni corrected P-value is below 0.002)

Significant P-values are indicated using an asterisk.

Results are presented as median and range (minimum – maximum) or number (percentage)

**Additional file 1: Table S8. RXRA rs10881578 polymorphic variants and demographic and clinical data of HD patients (n = 872).**

| Parameter | AA | AG | GG | Mode  of inheritance | Odds ratio (95% CI) | P value |
| --- | --- | --- | --- | --- | --- | --- |
|  | n = 447 | n = 339 | n = 86 |  |  |  |
| Male gender, n, % of all | 238 (53.2) | 200 (59.0) | 50 (58.1) | GG vs AA + AG | 1.104 (0.703, 1.732) | 0.754^b^ |
|  |  |  |  | AG + GG vs AA | 1.255 (0.960, 1.640) | 0.112^b^ |
|  |  |  |  | GG vs AA | 0.820 (0.514, 1.308) | 0.474^b^ |
| Age, years | 67.1 (17.1 - 93.4) | 66.7 (17.9 - 95.9) | 67.2 (36.6 - 91.9) | GG vs AA + AG |  | 0.226^a^ |
|  |  |  |  | AG + GG vs AA |  | 0.913^a^ |
|  |  |  |  | GG vs AA |  | 0.292^a^ |
| RRT vintage, years | 5.6 (0.1 - 28) | 5.9 (0.2 - 28.3) | 5.5 (0.8 - 15.8) | GG vs AA + AG |  | 0.841^a^ |
|  |  |  |  | AG + GG vs AA |  | 0.410^a^ |
|  |  |  |  | GG vs AA |  | 0.703^a^ |
| Diabetic nephropathy, n, % of all | 143 (32.0) | 81 (23.9) | 23 (26.7) | GG vs AA + AG | 0.916 (0.554, 1.513) | 0.828^b^ |
|  |  |  |  | AG + GG vs AA | 0.689 (0.512, 0.927) | 0.017^b,c^ |
|  |  |  |  | GG vs AA | 1.288 (0.768, 2.161) | 0.404^b^ |
| Hypertensive nephropathy, n, % of all | 89 (19.9) | 68 (20.1) | 21 (24.4) | GG vs AA + AG | 1.294 (0.768, 2.182) | 0.407^b^ |
|  |  |  |  | AG + GG vs AA | 1.065 (0.766, 1.481) | 0.769 ^b^ |
|  |  |  |  | GG vs AA | 0.769 (0.447, 1.326) | 0.420^b^ |
| Coronary artery disease, n, % of all | 167 (37.4) | 116 (34.2) | 36 (41.9) | GG vs AA + AG | 1.280 (0.814, 2.012) | 0.341^b^ |
|  |  |  |  | AG + GG vs AA | 0.934 (0.709, 1.23) | 0.776^b^ |
|  |  |  |  | GG vs AA | 0.828 (0.518, 1.324)) | 0.506^b^ |
| Myocardial infarction, n, % of all | 91 (20.4) | 68 (20.1) | 25 (29.1) | GG vs AA + AG | 1.616 (0.983, 2.656) | 0.077^b^ |
|  |  |  |  | AG + GG vs AA | 1.096 (0.791, 1.517) | 0.639^b^ |
|  |  |  |  | GG vs AA | 0.624 (0.371, 1.048) | 0.099^b^ |

a - Mann Whitney test

b - Chi - square test with Yates correction

c – non-significant after the Bonferroni correction (for 1 SNP, 3 inheritance models, and 7 phenotypes, a significant Bonferroni corrected P-value is below 0.002)

Results are presented as median and range (minimum – maximum) or number (percentage)

## **Additional file 1: Table S9. *LXRA* rs2279238 polymorphic variants and demographic and clinical data of HD patients (n = 861).**

| Parameter | GG | AG | AA | Mode  of inheritance | Odds ratio (95% CI) | P value |
| --- | --- | --- | --- | --- | --- | --- |
|  | n = 595 | n = 241 | n = 25 |  |  |  |
| Male gender, n, % of all | 354 (58.0) | 123 (51.0) | 16 (64.0) | AA vs GG + AG | 1.338 (0.585, 3.063) | 0.627^b^ |
|  |  |  |  | AG + AA vs GG | 0.745 (0.557, 0.997) | 0.056^b^ |
|  |  |  |  | AA vs GG | 1.21 (0.526, 2.784) | 0.809^b^ |
| Age, years | 61 (14.4 - 91) | 60.7 (11.1 - 85.5) | 62.2 (35.2 - 83.3) | AA vs GG + AG |  | 0.785^a^ |
|  |  |  |  | AG + AA vs GG |  | 0.882^a^ |
|  |  |  |  | AA vs GG |  | 0.780^a^ |
| RRT vintage, years | 5.7 (0.1 - 28) | 5.9 (0.2 - 28.3) | 5.1 (0.2 - 13.6) | AA vs GG + AG |  | 0.588^a^ |
|  |  |  |  | AG + AA vs GG |  | 0.694^a^ |
|  |  |  |  | AA vs GG |  | 0.615^a^ |
| Diabetic nephropathy, n, % of all | 167 (28.1) | 70 (29.0) | 8 (32.0) | AA vs GG + AG | 1.189 (0.506, 2.793) | 0.862^b^ |
|  |  |  |  | AG + AA vs GG | 1.063 (0.773, 1.463) | 0.767^b^ |
|  |  |  |  | AA vs GG | 1.206 (0.511, 2.848) | 0.841^b^ |
| Hypertensive nephropathy, n, % of all | 121 (20.3) | 47 (19.5) | 7 (28.0) | AA vs GG + AG | 1.546 (0.635, 3.763) | 0.474^b^ |
|  |  |  |  | AG + AA vs GG | 0.998 (0.697, 1.429) | 1.000^b^ |
|  |  |  |  | AA vs GG | 1.523 (0.622, 3.73) | 0.500^b^ |
| Coronary artery disease, n, % of all | 210 (35.3) | 94 (39.0) | 12 (48.0) | AA vs GG + AG | 1.615 (0.728, 3.585) | 0.328^b^ |
|  |  |  |  | AG + AA vs GG | 1.215 (0.902, 1.635) | 0.228^b^ |
|  |  |  |  | AA vs GG | 1.692 (0.759, 3.776) | 0.278^b^ |
| Myocardial infarction, n, % of all | 130 (21.8) | 44 (18.3) | 8 (32.0) | AA vs GG + AG | 1.79 (0.76, 4.217) | 0.271^b^ |
|  |  |  |  | AG + AA vs GG | 0.869 (0.606, 1.246) | 0.501^b^ |
|  |  |  |  | AA vs GG | 1.683 (0.71, 3.988) | 0.342^b^ |

a - Mann Whitney test

b - Chi - square test with Yates correction

Results are presented as median and range (minimum – maximum) or number (percentage)

## **Additional file 1: Table S10. *LXRA* rs7120118 polymorphic variants and demographic and clinical data of HD patients (n = 860).**

| Parameter | TT | CT | CC | Mode  of inheritance | Odds ratio (95% CI) | P value |
| --- | --- | --- | --- | --- | --- | --- |
|  | n = 409 | n = 375 | n = 76 |  |  |  |
| Male gender, n, % of all | 241 (58.9) | 202 (53.9) | 40 (52.6) | CC vs TT + CT | 0.855 (0.534, 1.371) | 0.597^b^ |
|  |  |  |  | CT + CC vs TT | 0.807 (0.616, 1.058) | 0.137^b^ |
|  |  |  |  | CC vs TT | 0.775 (0.474, 1.266) | 0.371^b^ |
| Age, years | 61.4 (14.4 - 88.2) | 60.7 (11.1 - 91) | 60.5 (13.7 - 83.3) | CC vs TT + CT |  | 0.268^a^ |
|  |  |  |  | CT + CC vs TT |  | 0.891^a^ |
|  |  |  |  | CC vs TT |  | 0.309^a^ |
| RRT vintage, years | 5.7 (0.1 - 28) | 5.7 (0.1 - 28.3) | 6 (0.2 - 21.1) | CC vs TT + CT |  | 0.368^a^ |
|  |  |  |  | CT + CC vs TT |  | 0.806^a^ |
|  |  |  |  | CC vs TT |  | 0.395^a^ |
| Diabetic nephropathy, n, % of all | 107 (26.2) | 110 (29.3) | 27 (35.5) | CC vs TT + CT | 1.44 (0.878, 2.362) | 0.188^b^ |
|  |  |  |  | CT + CC vs TT | 1.231 (0.914, 1.659) | 0.196^b^ |
|  |  |  |  | CC vs TT | 1.555 (0.926, 2.613) | 0.124^b^ |
| Hypertensive nephropathy, n, % of all | 85 (20.8) | 75 (20.0) | 15 (19.7) | CC vs TT + CT | 0.959 (0.531, 1.732) | 1.000^b^ |
|  |  |  |  | CT + CC vs TT | 0.95 (0.682, 1.325) | 0.829^b^ |
|  |  |  |  | CC vs TT | 0.937 (0.508, 1.731) | 0.958^b^ |
| Coronary artery disease, n, % of all | 140 (34.2) | 141 (47.6) | 35 (46.1) | CC vs TT + CT | 1.528 (0.951, 2.455) | 0.101^b^ |
|  |  |  |  | CT + CC vs TT | 1.23 (0.931, 1.625) | 0.166^b^ |
|  |  |  |  | CC vs TT | 1.64 (1, 2.691) | 0.066^b^ |
| Myocardial infarction, n, % of all | 78 (19.1) | 79 (21.1) | 25 (32.9) | CC vs TT + CT | 1.958 (1.176, 3.258) | 0.013^b,c^ |
|  |  |  |  | CT + CC vs TT | 1.272 (0.914, 1.769) | 0.178^b^ |
|  |  |  |  | CC vs TT | 2.08 (1.214, 3.564) | 0.011^b,c^ |

a - Mann Whitney test

b - Chi - square test with Yates correction

c – non-significant after the Bonferroni correction (for 1 SNP, 3 inheritance models, and 7 phenotypes, a significant Bonferroni corrected P-value is below 0.002)

Results are presented as median and range (minimum – maximum) or number (percentage)

## **Additional file 1: Table S11. *LXRA* rs11039155 polymorphic variants and demographic and clinical data of HD patients (n = 861).**

| Parameter | GG | AG | AA | Mode  of inheritance | Odds ratio (95% CI) | P value |
| --- | --- | --- | --- | --- | --- | --- |
|  | n = 604 | n = 232 | n = 25 |  |  |  |
| Male gender, n, % of all | 352 (58.3) | 117 (50.4) | 14 (56.0) | AA vs GG + AG | 0.996 (0.447, 2.22) | 1.000^b^ |
|  |  |  |  | AG + AA vs GG | 0.744 (0.555, 0.998) | 0.057^b^ |
|  |  |  |  | AA vs GG | 0.911 (0.407, 2.04) | 0.985^b^ |
| Age, years | 60.7 (14.4 - 91) | 61.2 (11.1 - 85.5) | 63 (35.2 - 83.3) | AA vs GG + AG |  | 0.945^a^ |
|  |  |  |  | AG + AA vs GG |  | 0.729^a^ |
|  |  |  |  | AA vs GG |  | 0.979^a^ |
| RRT vintage, years | 5.7 (0.1 - 28) | 5.7 (0.2 - 28.3) | 6.1 (0.2 - 13.6) | AA vs GG + AG |  | 1.000^a^ |
|  |  |  |  | AG + AA vs GG |  | 0.634^a^ |
|  |  |  |  | AA vs GG |  | 0.978^a^ |
| Diabetic nephropathy, n, % of all | 171 (28.3) | 67 (28.9) | 7 (28.0) | AA vs GG + AG | 0.977 (0.403, 2.37) | 1.000^b^ |
|  |  |  |  | AG + AA vs GG | 1.024 (0.741, 1.414) | 0.951^b^ |
|  |  |  |  | AA vs GG | 0.985 (0.404, 2.4) | 1.000^b^ |
| Hypertensive nephropathy, n, % of all | 121 (20.0) | 47 (20.3) | 6 (24.0) | AA vs GG + AG | 1.256 (0.494, 3.193) | 0.821^b^ |
|  |  |  |  | AG + AA vs GG | 1.037 (0.722, 1.489) | 0.917^b^ |
|  |  |  |  | AA vs GG | 1.261 (0.493, 3.225) | 0.818^b^ |
| Coronary artery disease, n, % of all | 214 (35.4) | 90 (38.8) | 11 (44.0) | AA vs GG + AG | 1.375 (0.616, 3.067) | 0.568^b^ |
|  |  |  |  | AG + AA vs GG | 1.18 (0.873, 1.594) | 0.317^b^ |
|  |  |  |  | AA vs GG | 1.432 (0.639, 3.21) | 0.507^b^ |
| Myocardial infarction, n, % of all | 131 (21.7) | 43 (18.5) | 8 (32.0) | AA vs GG + AG | 1.79 (0.76, 4.217) | 0.271^b^ |
|  |  |  |  | AG + AA vs GG | 0.894 (0.622, 1.284) | 0.606^b^ |
|  |  |  |  | AA vs GG | 1.699 (0.717, 4.025) | 0.331^b^ |

a - Mann Whitney test

b - Chi - square test with Yates correction

Results are presented as median and range (minimum – maximum) or number (percentage)

|  |  |  |  |  |  | Myocardial infarction |  |  |
| --- | --- | --- | --- | --- | --- | --- | --- | --- |
|  | Age | RRT duration | Diabetic nephropathy | Hypertensive nephropathy | Coronary artery disease |  |  |  |
|  |  |  |  |  |  |  |  |  |
| Male gender | 0.003^a*^  -3.04 ± 1.00^d^ |  |  |  | 0.002^b*^  1.55 (1.17 - 2.06)^e^ | 0.0005^b*^  1.83 (1.30 - 2.57)^e^ |  |  |
|  |  | 0.306^a^ | 0.593^b^ | 0.484^b^ |  |  |  |  |
|  |  |  |  |  |  |  |  |  |
| Age |  |  | 0.002^a*^  3.39 ± 1.10^d^ | 0.011^a*^  3.78 ± 1.24^d^ | 9.0 E-13^a*^  8.00 ± 1.00^d^ | 5.2 E-6^a*^  6.31 ± 1.21^d^ |  |  |
|  |  | 0.142^c^ |  |  |  |  |  |  |
|  |  |  |  |  |  |  |  |  |
| RRT duration |  |  | 0.0001^a*^  3.39 ± 1.10^d^ | 0.075^a^ | 0.055^a^ | 0.220^a^ |  |  |
|  |  |  |  |  |  |  |  |  |
|  |  |  |  |  |  |  |  |  |
| Diabetic nephropathy |  |  |  |  | ≈0^b*^  2.21 (1.63 - 2.98)^e^ | 0.0002^b*^  1.90 (1.35 - 2.68)^e^ |  |  |
| Hypertensive nephropathy |  |  |  |  | 0.048^b*^  1.40 (1.00 - 1.96)^e^ | 0.039^b*^  1.49 (1.02 - 2.19)^e^ |  |  |

## **Additional file 1: Table S12. The association structure among selected phenotypes of hemodialysis patients.**

a – Mann-Whitney U test P-value; b – Chi-squared test P-value; c – Spearman rank order correlations P-value; d – β coefficient ± standard error in univariate linear regression; e – Odds ratio ± 95% confidence intervals

Significant P-values are indicated using an asterisk.

## **Additional file 1: Table S13. All-cause mortality since the start of 7.5-year prospective study in 440 HD patients stratified by selected phenotypes.**

| **All-cause mortality since the start of 7.5-year prospective study** | | | | | |
| --- | --- | --- | --- | --- | --- |
| Phenotype | Log-rank P value | HR (95% CI) | HR P value | Adjusted HR (95% CI)^a^ | Adjusted HR P value |
| Dyslipidemia by K/DOQI criteria,  n = 137 | 0.194 | 0.867 (0.698 - 1.076) | 0.194 | 0.890 (0.676 - 1.171) | 0.405 |
| Atherogenic dyslipidemia, n = 123 | 0.592 | 1.065 (0.846 - 1.340) | 0.591 | 0.936 (0.697 - 1.257) | 0.659 |
| Coronary artery disease, n = 146 | <0.00001* | 2.026 (1.629 - 2.519) | <0.000001* | 1.573 (1.161 - 2.130) | 0.003* |
| Myocardial infarction, n = 88 | <0.00001* | 1.773 (1.401 - 2.242) | 0.000002* | 1.499 (1.000 - 2.246) | 0.050 |
| Diabetic nephropathy, n = 96 | 0.00008* | 1.602 (1.273 - 2.016) | 0.00006* | 1.437 (1.068 - 1.935) | 0.017* |

a - adjustment for age, gender, renal replacement therapy duration prior to the start of the study, body mass index, coronary artery disease, and diabetic nephropathy, as appropriate

n – number of deceased suffering from a given reason

Significant P-values are indicated using an asterisk.

## **Additional file 1: Table S14. Associations between *ENHO* rs2281997 polymorphic variants and LDL-cholesterol levels ≥100 mg/dL in hemodialysis patients.**

| Genotypes and MAF | LDL-cholesterol ≥ 100 mg/dL  n = 400 | LDL-cholesterol <100 mg/dL  n = 473 | Odds ratio (95% CI) | P value^a^ | P*_trend_*^b^ | P*_genotype_*^a^ |
| --- | --- | --- | --- | --- | --- | --- |
|  | n (% of all) | n (% of all) |  |  |  |  |
| CC | 185 (46.3) | 261 (55.2) | Reference | - | 0.0007* | 0.002* |
| CT | 170 (42.5) | 186 (39.3) | 1.289 (0.974 - 1.707) | 0.076 |  |  |
| TT | 45 (11.3) | 26 (5.5) | 2.442 (1.454 - 4.100) | 0.0006* |  |  |
| TT vs CC + CT | | | 2.179 (1.318 - 3.602) | 0.002* |  |  |
| CT + TT vs CC | | | 1.431 (1.095 -1.869) | 0.009^c^ |  |  |
| MAF | 260 (32.5) | 238 (25.2) | 1.432 (1.163 - 1.764) | 0.0007* |  |  |
| P for HWE | 0.531 | 0.336 |  |  |  |  |

a - Chi^2^ test; b – Chi^2^ Test for Trend in Proportions; c – not significant after the Bonferroni correction (for 1 SNP, 7 analyzes for the association, and 1 phenotype, a significant Bonferroni corrected P-value is below 0.007)

Significant P-values are indicated using an asterisk.

## **Additional file 1: Table S15. Associations between *ENHO* rs2281997 polymorphic variants and non-HDL-cholesterol levels ≥130 mg/dL in hemodialysis patients (n = 873).**

| Genotypes and MAF | Non-HDL-cholesterol  ≥ 130 mg/dL  n = 418 | Non-HDL-cholesterol  < 130 mg/dL  n = 455 | Odds ratio (95% CI) | P value^a^ | P*_trend_*^b^ | P*_genotype_*^a^ |
| --- | --- | --- | --- | --- | --- | --- |
|  | n (% of all) | n (% of all) |  |  |  |  |
| CC | 202 (48.3) | 244 (53.6) | Reference | - | 0.123 | 0.282 |
| CT | 179 (42.8) | 177 (38.9) | 1.222 (0.924 - 1.615) | 0.160 |  |  |
| TT | 37 (8.9) | 34 (7.5) | 1.315 (0.796 - 2.171) | 0.284 |  |  |
| TT vs CC + CT | | | 1.202 (0.740 - 1.955) | 0.456 |  |  |
| CT + TT vs CC | | | 1.237 (0.948 - 1.613) | 0.118 |  |  |
| MAF | 253 (30.3) | 245 (26.9) | 1.178 (0.957 - 1.450) | 0.123 |  |  |
| P for HWE | 0.766 | 0.808 |  |  |  |  |

a - Chi^2^ test; b – Chi^2^ Test for Trend in Proportions

## **Additional file 1: Table S16. Associations between *ENHO* rs2281997 polymorphic variants and TG ≥ 200 mg/dL in hemodialysis patients (n = 873).**

| Genotypes and MAF | TG ≥ 200 mg/dL n = 204 | TG < 200 mg/dL  n = 669 | Odds ratio (95% CI) | P value^a^ | P*_trend_*^b^ | P*_genotype_*^a^ |
| --- | --- | --- | --- | --- | --- | --- |
|  | n (% of all) | n (% of all) |  |  |  |  |
| CC | 106 (52.0) | 340 (50.8) | Reference | - | 0.241 | 0.078 |
| CT | 89 (43.6) | 267 (39.9) | 1.069 (0.773 - 1.479) | 0.686 |  |  |
| TT | 9 (4.4) | 62 (9.3) | 0.466 (0.224 - 0.968) | 0.037^c^ |  |  |
| TT vs CC + CT | | | 0.452 (0.221 - 0.926) | 0.026^c^ |  |  |
| CT + TT vs CC | | | 0.955 (0.698 - 1.308) | 0.776 |  |  |
| MAF | 107 (26.2) | 391 (29.2) | 0.861 (0.671 - 1.106) | 0.241 |  |  |
| P for HWE | 0.069 | 0.363 |  |  |  |  |

a - Chi^2^ test; b – Chi^2^ Test for Trend in Proportions; c – not significant after the Bonferroni correction (for 1 SNP, 7 analyzes for the association, and 1 phenotype, a significant Bonferroni corrected P-value is below 0.007)

## **Additional file 1: Table S17. Associations between *ENHO* rs2281997 and *ENHO* rs72735260 polymorphic variants and dyslipidemia diagnosed by K/DOQI recommendations in hemodialysis patients.**

| Genotypes and MAF | Dyslipidemic patients | Non-dyslipidemic patients by K/DOQI | Odds ratio (95% CI) | P value^a^ | P*_trend_*^b^ | P*_genotype_*^a^ |
| --- | --- | --- | --- | --- | --- | --- |
|  | n (% of all) | n (% of all) |  |  |  |  |
| ENHO rs2281997, n = 873, P for HWE = 0.997 | | | | | | |
| CC | 215 (46.8) | 231 (55.8) | Reference | - | 0.0002* | 0.0002* |
| CT | 191 (41.6) | 165 (39.9) | 1.244 (0.941 - 1.644) | 0.125 |  |  |
| TT | 53 (11.5) | 18 (4.3) | 3.164 (1.796 - 5.572) | 0.00003* |  |  |
| TT vs CC + CT | | | 2.872 (1.653 – 4.989) | 0.0001* |  |  |
| CT + TT vs CC | | | 1.433 (1.097 – 1.871) | 0.008^c^ |  |  |
| MAF | 297 (32.4) | 201 (24.3) | 1.492 (1.209 – 1.842) | 0.0002* |  |  |
| P for HWE | 0.291 | 0.087 |  |  |  |  |
| ENHO rs72735260, n = 848, P for HWE = 0.681 | | | | | | |
| GG | 332 (74.8) | 297 (73.5) | Reference | - | 0.771 | 0.849 |
| GT | 102 (23.0) | 99 (24.5) | 0.922 (0.671 - 1.266) | 0.615 |  |  |
| TT | 10 (2.3) | 8 (2.0) | 1.118 (0.436 - 2.871) | 0.816 |  |  |
| TT vs GG + GT | | | 1.141 (0.446 - 2.919) | 0.784 |  |  |
| GT + TT vs GG | | | 0.936 (0.688 - 1.274) | 0.676 |  |  |
| MAF | 122 (13.7) | 115 (14.2) | 0.960 (0.729 - 1.263) | 0.769 |  |  |
| P for HWE | 0.517 | 0.940 |  |  |  |  |

a - Chi^2^ test; b – Chi^2^ Test for Trend in Proportions; c – not significant after the Bonferroni correction (for 2 SNPs, 7 analyzes for the association in each SNP, and 1 phenotype, a significant Bonferroni corrected P-value is below 0.004)

Significant P-values are indicated using an asterisk.

## **Additional file 1: Table S18. Associations between *ENHO* rs2281997 polymorphic variants and hyper-LDL-cholesterolemic pattern of dyslipidemia in hemodialysis patients.**

| Genotypes and MAF | Patients with hyper-LDL-cholesterolemic pattern of dyslipidemia  n = **267** | Non-dyslipidemic patients  n = 414 | Odds ratio (95% CI) | P value^a^ | P*_trend_*^b^ | P*_genotype_*^a^ |
| --- | --- | --- | --- | --- | --- | --- |
|  | n (% of all) | n (% of all) |  |  |  |  |
| CC | 120 (44.9) | 231 (55.8) | Reference | - | 0.0001^*^ | 0.0001^*^ |
| CT | 114 (42.7) | 165 (39.9) | 1.330 (0.961 - 1.841) | 0.085 |  |  |
| TT | 33 (12.4) | 18 (4.3) | 3.529 (1.908 - 6.529) | 0.00003^*^ |  |  |
| TT vs CC + CT | | | 3.103 (1.709 - 5.634) | 0.0001^*^ |  |  |
| CT + TT vs CC | | | 1.546 (1.135 - 2.107) | 0.006^*^ |  |  |
| MAF | 180 (33.7) | 201 (24.3) | 1.586 (1.248 - 2.016) | 0.0002^*^ |  |  |
| P for HWE | 0.466 | 0.087 |  |  |  |  |

a - Chi^2^ test; b – Chi^2^ Test for Trend in Proportions

Significant P-values are indicated using an asterisk. All these values are significant after the Bonferroni correction (for 1 SNP, 7 analyzes for the association, and 1 phenotype, a significant Bonferroni corrected P-value is below 0.007)

## **Additional file 1: Table S19. Associations between *ENHO* rs2281997 polymorphic variants and simultaneously occurring hyper-TG/hyper-non-HDL-cholesterolemic and hyper-LDL-cholesterolemic patterns of dyslipidemia in hemodialysis patients.**

| Genotypes and MAF | Dyslipidemic patients  n = 123 | Non-dyslipidemic patients  n = 414 | Odds ratio (95% CI) | P value^a^ | P*_trend_*^b^ | P*_genotype_*^a^ |
| --- | --- | --- | --- | --- | --- | --- |
|  | n (% of all) | n (% of all) |  |  |  |  |
| CC | 63 (51.2) | 231 (55.8) | Reference | - | 0.393 | 0.669 |
| CT | 54 (43.9) | 165 (39.9) | 1.200 (0.793 - 1.817) | 0.389 |  |  |
| TT | 6 (4.9) | 18 (4.3) | 1.222 (0.466 - 3.208) | 0.683 |  |  |
| TT vs CC + CT | | | 1.128 (0.438 - 2.908) | 0.803 |  |  |
| CT + TT vs CC | | | 1.202 (0.803 - 1.799) | 0.370 |  |  |
| MAF | 66 (26.8) | 201 (24.3) | 1.144 (0.8275 - 1.581) | 0.416 |  |  |
| P for HWE | 0.190 | 0.087 |  |  |  |  |

a - Chi^2^ test; b – Chi^2^ Test for Trend in Proportions

## **Additional file 1: Table S20. Associations between *ENHO* rs2281997 polymorphic variants and hyper-TG/hyper-non-HDL-cholesterolemic pattern of dyslipidemia in hemodialysis patients.**

| Genotypes and MAF | Dyslipidemic patients  n = 47 | Non-dyslipidemic patients  n = 414 | Odds ratio (95% CI) | P value | P*_trend_*^b^ | P*_genotype_*^a^ |
| --- | --- | --- | --- | --- | --- | --- |
|  | n (% of all) | n (% of all) |  |  |  |  |
| CC | 25 (53.2) | 231 (55.8) | Reference | - | 0.606 | 0.803 |
| CT | 19 (40.4) | 165 (39.9) | 1.064 (0.567 - 1.996) | 0.847^a^ |  |  |
| TT | 3 (6.4) | 18 (4.3) | 1.540 (0.424 - 5.595) | 0.456^c^ |  |  |
| TT vs CC + CT | | | 1.500 (0.425 - 5.295) | 0.463^c^ |  |  |
| CT + TT vs CC | | | 1.111 (0.607 - 2.034) | 0.733^a^ |  |  |
| MAF | 25 (26.6) | 201 (24.3) | 1.130 (0.696 - 1.834) | 0.620^a^ |  |  |
| P for HWE | 0.808 | 0.087 |  |  |  |  |

a - Chi^2^ test; b - Chi^2^ Test for Trend in Proportions; c - Fisher exact test

## **Additional file 1: Table S21. Distribution of ENHO polymorphic variants in HD patients categorized by atherogenic dyslipidemia.**

| Genotypes and MAF | Atherogenic dyslipidemia | Without atherogenic dyslipidemia | Odds ratio (95% CI) | P value^a^ | P*_trend_*^b^ | P*_genotype_*^a^ |
| --- | --- | --- | --- | --- | --- | --- |
|  | n (% of all) | n (% of all) |  |  |  |  |
| ENHO rs2281997, n = 873, P for HWE = 0.997 | | | | | | |
| CC | 255 (56.2) | 191 (45.6) | Reference | - | 0.0007^*^ | 0.003^*^ |
| CT | 171 (37.7) | 185 (44.2) | 0.692 (0.523 - 0.916) | 0.010^c^ |  |  |
| TT | 28 (6.2) | 43 (10.3) | 0.488 (0.292 - 0.814) | 0.005^*^ |  |  |
| TT vs CC + CT | | | 0.575 (0.350 - 0.944) | 0.027^c^ |  |  |
| CT + TT vs CC | | | 0.654 (0.501 - 0.854) | 0.002^*^ |  |  |
| MAF | 227 (25.0) | 271 (32.3) | 0.697 (0.566 - 0.859) | 0.0007^*^ |  |  |
| P for HWE | 0.925 | 0.855 |  |  |  |  |
| ENHO rs72735260, n = 848, P for HWE = 0.681 | | | | | | |
| GG | 329 (74.8) | 300 (73.5) | Reference | - | 0.784 | 0.836 |
| GT | 101 (23.0) | 100 (24.5) | 0.921 (0.670 - 1.265) | 0.612 |  |  |
| TT | 10 (2.3) | 8 (2.0) | 1.140 (0.444 - 2.926) | 0.785 |  |  |
| TT vs GG + GT | | | 1.163 (0.454 - 2.976) | 0.753 |  |  |
| GT + TT vs GG | | | 0.937 (0.689 - 1.275) | 0.679 |  |  |
| MAF | 121 (13.8) | 116 (14.2) | 0.962 (0.731 - 1.266) | 0.782 |  |  |
| P for HWE | 0.499 | 0.921 |  |  |  |  |

a - Chi^2^ test; b – Chi^2^ Test for Trend in Proportions; c – not significant after the Bonferroni correction (for 2 SNPs, 7 analyzes for the association in each SNP, and 1 phenotype, a significant Bonferroni corrected P-value is below 0.004)

Significant P-values are indicated using an asterisk. All these values are significant after the Bonferroni correction (for 2 SNPs, 7 analyzes for the association in each SNP, and 1 phenotype, a significant Bonferroni corrected P-value is below 0.004)

# Additional file 1: Table S22. Mortality of HD patients (n = 440) by the Kaplan-Meier analysis with P values obtained in the subsequent log-rank test for models of inheritance as well as multiple P values obtained in comparisons of mortality in patients separated by genotypes.

| Inheritance model | Dominant | Recessive | Additive | Genotypes |
| --- | --- | --- | --- | --- |
| All-cause mortality since the start of 7.5-year prospective study, n = 280 | | | | |
| *ENHO* rs2281997 | 0.940 | 0.448 | 0.518 | 0.719 |
| ENHO rs72735260 | 0.882 | 0.691 | 0.701 | 0.876 |
| RXRA rs749759 | 0.193 | 0.895 | 0.841 | 0.496 |
| RXRA rs10776909 | 0.480 | 0.987 | 0.936 | 0.785 |
| *RXRA* rs10881578 | 0.140 | 0.457 | 0.307 | 0.243 |
| *LXRA* rs2279238 | 0.046* | 0.190 | 0.142 | 0.122 |
| *LXRA* rs7120118 | 0.036* | 0.079 | 0.051 | 0.101 |
| *LXRA* rs11039155 | 0.018* | 0.144 | 0.095 | 0.063 |
| Cardiovascular mortality since the start of 7.5-year prospective study, n = 183 | | | | |
| *ENHO* rs2281997 | 0.492 | 0.534 | 0.416 | 0.898 |
| ENHO rs72735260 | 0.324 | 0.738 | 0.804 | 0.113 |
| RXRA rs749759 | 0.832 | 0.828 | 0.801 | 0.835 |
| RXRA rs10776909 | 0.906 | 0.754 | 0.883 | 0.753 |
| *RXRA* rs10881578 | 0.987 | 0.907 | 0.997 | 0.918 |
| *LXRA* rs2279238 | 0.147 | 0.555 | 0.469 | 0.316 |
| *LXRA* rs7120118 | 0.782 | 0.229 | 0.273 | 0.345 |
| *LXRA* rs11039155 | 0.153 | 0.885 | 0.939 | 0.212 |

Significant P-values are indicated using an asterisk.

## **Additional file 1: Table S23. Associations between RXRA polymorphic variants and dyslipidemia diagnosed by K/DOQI recommendations in hemodialysis patients.**

| Genotypes and MAF | Dyslipidemic patients | Non-dyslipidemic patients | Odds ratio (95% CI) | P value^a^ | P*_trend_*^b^ | P*_genotype_*^a^ |
| --- | --- | --- | --- | --- | --- | --- |
|  | n (% of all) | n (% of all) |  |  |  |  |
| RXRA rs749759, n = 868, P for HWE = 0.852 | | | | | | |
| GG | 235 (51.2) | 227 (55.5) | Reference | - | 0.142 | 0.332 |
| AG | 187 (40.7) | 157 (38.4) | 1.151 (0.870 - 1.522) | 0.326 |  |  |
| AA | 37 (8.1) | 25 (6.1) | 1.430 (0.834 - 2.451) | 0.192 |  |  |
| AA vs GG + AG | | | 1.347 (0.796 - 2.279) | 0.266 |  |  |
| AG + AA vs GG | | | 1.189 (0.910 - 1.553) | 0.205 |  |  |
| MAF | 261 (28.4) | 207 (25.3) | 1.173 (0.948 - 1.451) | 0.143 |  |  |
| P for HWE | 0.981 | 0.755 |  |  |  |  |
| RXRA rs10776909, n = 872, P for HWE = 0.869 | | | | | | |
| CC | 278 (60.6) | 268 (64.9) | Reference | - | 0.163 | 0.377 |
| CT | 158 (34.4) | 129 (31.2) | 1.181 (0.886 - 1.573) | 0.256 |  |  |
| TT | 23 (5.0) | 16 (3.9) | 1.386 (0.716 - 2.681) | 0.331 |  |  |
| TT vs CC + CT | | | 1.309 (0.682 - 2.513) | 0.417 |  |  |
| CT + TT vs CC | | | 1.203 (0.914 - 1.585) | 0.188 |  |  |
| MAF | 204 (22.2) | 161 (19.5) | 1.180 (0.936 - 1.488) | 0.162 |  |  |
| P for HWE | 0.928 | 0.923 |  |  |  |  |
| RXRA rs10881578, n = 872, P for HWE = 0.069 | | | | | | |
| AA | 231 (50.3) | 216 (52.3) | Reference | - | 0.836 | 0.633 |
| AG | 185 (40.3) | 154 (37.3) | 1.123 (0.846 - 1.491) | 0.421 |  |  |
| GG | 43 (9.4) | 43 (10.4) | 0.935 (0.589 - 1.484) | 0.776 |  |  |
| GG vs AA + AG | | | 0.889 (0.570 - 1.388) | 0.606 |  |  |
| AG + GG vs AA | | | 1.082 (0.829 -1.412) | 0.561 |  |  |
| MAF | 271 (29.5) | 240 (29.1) | 1.023 (0.832 - 1.257) | 0.831 |  |  |
| P for HWE | 0.501 | 0.052 |  |  |  |  |

a - Chi^2^ test; b – Chi^2^ Test for Trend in Proportions

## **Additional file 1: Table S24. Associations between RXRA polymorphic variants and atherogenic dyslipidemia in hemodialysis patients.**

| Genotypes and MAF | Atherogenic dyslipidemia | Without atherogenic dyslipidemia | Odds ratio (95% CI) | P value^a^ | P*_trend_*^b^ | P*_genotype_*^a^ |
| --- | --- | --- | --- | --- | --- | --- |
|  | n (% of all) | n (% of all) |  |  |  |  |
| RXRA rs749759, n = 868, P for HWE = 0.852 | | | | | | |
| GG | 240 (53.0) | 222 (53.5) | Reference | - | 0.979 | 0.904 |
| AG | 182 (40.2) | 162 (39.0) | 1.039 (0.786 - 1.374) | 0.788 |  |  |
| AA | 31 (6.8) | 31 (7.5) | 0.925 (0.544 - 1.572) | 0.773 |  |  |
| AA vs GG + AG | | | 0.910 (0.543 - 1.525) | 0.720 |  |  |
| AG + AA vs GG | | | 1.021 (0.782 - 1.333) | 0.880 |  |  |
| MAF | 244 (26.9) | 224 (27.0) | 0.997 (0.807 - 1.233) | 0.979 |  |  |
| P for HWE | 0.658 | 0.847 |  |  |  |  |
| RXRA rs10776909, n = 872, P for HWE = 0.869 | | | | | | |
| CC | 296 (65.2) | 250 (59.8) | Reference | - | 0.157 | 0.242 |
| CT | 138 (30.4) | 149 (35.6) | 0.782 (0.588 - 1.042) | 0.092 |  |  |
| TT | 20 (4.4) | 19 (4.5) | 0.889 (0.464 - 1.703) | 0.723 |  |  |
| TT vs CC + CT | | | 0.968 (0.509 -1.840) | 0.920 |  |  |
| CT + TT vs CC | | | 0.794 (0.604 - 1.046) | 0.100 |  |  |
| MAF | 178 (19.6) | 187 (22.4) | 0.846 (0.672 - 1.066) | 0.156 |  |  |
| P for HWE | 0.447 | 0.590 |  |  |  |  |
| RXRA rs10881578, n = 872, P for HWE = 0.069 | | | | | | |
| AA | 227 (50.0) | 220 (52.6) | Reference | - | 0.613 | 0.663 |
| AG | 183 (40.3) | 156 (37.3) | 1.137 (0.857 - 1.509) | 0.374 |  |  |
| GG | 44 (9.7) | 42 (10.0) | 1.015 (0.640 - 1.611) | 0.949 |  |  |
| GG vs AA + AG | | | 0.961 (0.615 - 1.500) | 0.860 |  |  |
| AG + GG vs AA | | | 1.111 (0.852 - 1.450) | 0.437 |  |  |
| MAF | 271 (29.8) | 240 (28.7) | 1.056 (0.859 - 1.299) | 0.602 |  |  |
| P for HWE | 0.425 | 0.071 |  |  |  |  |

a - Chi^2^ test; b – Chi^2^ Test for Trend in Proportions

## **Additional file 1: Table S25. Associations between RXRA polymorphic variants and myocardial infarction (MI) in HD patients.**

| Genotypes and MAF | Patients  with MI | Patients  without MI | Odds ratio (95% CI) | P value^a^ | P*_trend_*^b^ | P*_genotype_*^a^ |
| --- | --- | --- | --- | --- | --- | --- |
|  | n (% of all) | n (% of all) |  |  |  |  |
| RXRA rs749759, n = 868, P for HWE = 0.852 | | | | | | |
| GG | 94 (51.6) | 368 (53.6) | Reference | - | 0.114 | 0.013^c^ |
| AG | 66 (36.3) | 278 (40.5) | 0.929 (0.654 - 1.320) | 0.683 |  |  |
| AA | 22 (12.1) | 40 (5.8) | 2.153 (1.221 - 3.798) | 0.007^c^ |  |  |
| AA vs GG + AG | | | 2.221 (1.283 - 3.842) | 0.004^c^ |  |  |
| AG + AA vs GG | | | 1.083 (0.781 - 1.503) | 0.631 |  |  |
| MAF | 110 (30.2) | 358 (26.1) | 1.227 (0.951 - 1.581) | 0.115 |  |  |
| P for HWE | 0.059 | 0.184 |  |  |  |  |
| RXRA rs10776909, n = 872, P for HWE = 0.869 | | | | | | |
| CC | 107 (58.2) | 439 (63.8) | Reference | - | 0.015^c^ | 0.002^c^ |
| CT | 60 (32.6) | 227 (33.0) | 1.084 (0.761 - 1.546) | 0.654 |  |  |
| TT | 17 (9.3) | 22 (3.2) | 3.170 (1.627 - 6.179) | 0.0004* |  |  |
| TT vs CC + CT | | | 3.082 (1.600 - 5.934) | 0.0004* |  |  |
| CT + TT vs CC | | | 1.269 (0.911 - 1.768) | 0.159 |  |  |
| MAF | 94 (25.5) | 271 (19.7) | 1.399 (1.069 - 1.831) | 0.014^c^ |  |  |
| P for HWE | 0.053 | 0.259 |  |  |  |  |
| RXRA rs10881578, n = 872, P for HWE = 0.069 | | | | | | |
| AA | 91 (49.5) | 356 (51.7) | Reference | - | 0.203 | 0.161 |
| AG | 68 (37.0) | 271 (39.4) | 0.982 (0.691 - 1.395) | 0.918 |  |  |
| GG | 25 (13.6) | 61 (8.9) | 1.603 (0.954 -2.695) | 0.073 |  |  |
| GG vs AA + AG | | | 1.616 (0.983 - 2.656) | 0.056 |  |  |
| AG + GG vs AA | | | 1.096 (0.792 - 1.517) | 0.581 |  |  |
| MAF | 118 (32.1) | 393 (28.6) | 1.181 (0.921 - 1.513) | 0.190 |  |  |
| P for HWE | 0.040* | 0.362 |  |  |  |  |

a - Chi^2^ test; b – Chi^2^ Test for Trend in Proportions, c – not significant after the Bonferroni correction (for 3 SNPs, 7 analyzes for the association in each SNP, and 1 phenotype, a significant Bonferroni corrected P-value is below 0.002)

Significant P-values are indicated using an asterisk. All these values (except for this for HWE) are significant after the Bonferroni correction (for 3 SNPs, 7 analyzes for the association in each SNP, and 1 phenotype, a significant Bonferroni corrected P-value is below 0.002)

## **Additional file 1: Table S26. Demographic, clinical and laboratory data of hemodialysis patients with myocardial infarction (MI) and without myocardial infarction (non-MI).**

| **Demographic data** | MI  N = 185 | Non-MI  N = 688 | P value |
| --- | --- | --- | --- |
| Male gender, n, % of all | 125 (67.6) | 324 (47.1) | 0.0005^b*^ |
| Age, years | 70.5 (38.9 - 92.3) | 64.3 (17.1 - 95.9) | 0.000007^a*^ |
| RRT duration, years | 5.8 (0.3 - 15.9) | 6.7 (0.1 - 28.3) | 0.195^a^ |
| **Clinical data** |  |  |  |
| Diabetic nephropathy, n, % of all | 73 (39.5) | 175 (25.4) | 0.0003* |
| Coronary artery disease, n, % of all | 177 (95.7) | 143 (20.8) | <0.000001^b*^ |
| BMI, kg/m^2^ | 26.6 (16.2 – 63.5) | 24.9 (12.8 – 59.2) | 0.0006^a*^ |
| BMI >30 kg/m^2^ (obesity), n, % of all | 38 (25.2) | 104 (18.0) | 0.062^b^ |
| **Type of RRT** |  |  |  |
| LF-HD, n, % of all | 95 (51.4) | 323 (46.9) | 0.326^b^ |
| PD as the first RRT modality, n, % of all | 6 (3.2) | 14 (2.0) | 0.514^b^ |
| **Laboratory data** |  |  |  |
| TC, mg/dL | 172.3 (51 - 363) | 178.4 (72 - 626) | 0.159^a^ |
| HDL-ch+olesterol, mg/dL | 40.7 (19 - 103) | 42.3 (6 - 103) | 0.109^a^ |
| Triglycerides, mg/dL | 174.3 (36.3 - 856) | 166.4 (29.8 - 691) | 0.133^a^ |
| LDL-cholesterol, mg/dL | 101.3 (13.3 - 350) | 103.7 (20 - 512) | 0.382^a^ |
| Non-HDL-cholesterol, mg/dL | 131.6 (32 - 282) | 136.2 (27 - 593) | 0.390^a^ |
| LDL-cholesterol/HDL-cholesterol ratio | 2.7 (0.5 - 10.9) | 2.7 (0.4 - 15.5) | 0.849^a^ |
| HDL-cholesterol/TC ratio | 0.25 (0.1 - 0.59) | 0.25 (0.05 - 0.64) | 0.984^a^ |
| TG/HDL-cholesterol ratio | 4.7 (0.6 - 17.5) | 4.6 (0.5 - 30.8) | 0.074^a^ |
| ALT, IU/L | 17.1 (2 - 131) | 16.9 (0.6 - 195) | 0.518^a^ |
| AST, IU/L | 17.6 (3 - 97) | 17.2 (4 - 152) | 0.210^a^ |
| GGT, IU/L | 53 (7 - 342) | 46.4 (1 - 682) | 0.199^a^ |
| ALP, U/L | 124.7 (25.8 - 1299.3) | 127 (24 - 1684) | 0.994^a^ |
| Adropin, ng/mL^c^ | 2.27 (1.03 - 5.48) | 2.07 (0.22 - 8.46) | 0.678 |
| PTH, pg/mL | 557 (7.3 - 3757) | 551.3 (12.9 - 3740.7) | 0.565^a^ |
| Ca, mg/dL | 8.9 (7.2 - 12.3) | 8.8 (5.4 - 12.8) | 0.573^a^ |
| P, mg/dL | 5 (1.8 - 10.5) | 5.2 (2 - 11.3) | 0.115^a^ |

a - Mann Whitney test

b - Chi - square test with Yates correction

Significant P-values are indicated using an asterisk.

## **Additional file 1: Table S27. Distribution of *RXRA* haplotypes in HD patients with myocardial infarction (cases) and HD patients without myocardial infarction (controls).**

| **Gene** | **Polymorphisms** | **Haplotype** | **Freq.** | **Case,Control Frequencies** | **Chi Square** | **P Value** | **P_corr_ Value^a^** | **OR (95% CI), p value^b^** | **OR (95% CI), p value^c^** |
| --- | --- | --- | --- | --- | --- | --- | --- | --- | --- |
| ***RXRA*** | **rs10881578_rs10776909** | AC | 0.620 | 0.570, 0.633 | 4.968 | 0.026 | 0.065 | **Reference** | 0.769 (0.609 - 0.971), 0.0269 |
|  |  | GC | 0.171 | 0.176, 0.169 | 0.088 | 0.767 | 0.984 | 1.150 (0.841 - 1.574), 0.3810 | 1.044 (0.771 - 1.412), 0.7821 |
|  |  | GT | 0.122 | 0.146, 0.116 | 2.408 | 0.121 | 0.298 | 1.400 (0.993 - 1.974), 0.0538 | 1.306 (0.936 - 1.821), 0.1150 |
|  |  | AT | 0.087 | 0.108, 0.081 | 2.683 | 0.101 | 0.249 | 1.473 (0.996 - 2.178), 0.0513 | 1.366 (0.933 - 1.999), 0.1074 |
|  | **rs10776909_rs749759** | CG | 0.675 | 0.626, 0.689 | 5.207 | 0.023 | 0.065 | **Reference** | 0.761 (0.599 - 0.967), 0.0250 |
|  |  | TA | 0.154 | 0.178, 0.147 | 2.110 | 0.146 | 0.368 | 1.326 (0.970 - 1.812), 0.0765 | 1.252 (0.923 - 1.699), 0.1476 |
|  |  | CA | 0.115 | 0.120, 0.114 | 0.095 | 0.758 | 0.980 | 1.143 (0.794 - 1.644), 0.4720 | 1.046 (0.733 - 1.494), 0.8035 |
|  |  | TG | 0.055 | 0.076, 0.050 | 3.803 | 0.051 | 0.135 | 1.679 (1.057 - 2.668), 0.0268 | 1.572 (0.997 - 2.481), 0.0500 |
|  | **rs10881578_rs10776909_rs749759** | ACG | 0.517 | 0.472, 0.529 | 3.843 | 0.050 | 0.205 | **Reference** | 0.799 (0.635 - 1.055), 0.0551 |
|  |  | GCG | 0.159 | 0.154, 0.160 | 0.064 | 0.800 | 1.000 | 1.076 (0.770 - 1.504), 0.6667 | 0.955 (0.696 - 1.312), 0.7770 |
|  | * | ACA | 0.103 | 0.099, 0.104 | 0.112 | 0.738 | 1.000 | 1.039 (0.696 - 1.551), 0.8532 | 0.921 (0.627 - 1.353), 0.6736 |
|  |  | GTA | 0.088 | 0.098, 0.086 | 0.557 | 0.456 | 0.978 | 1.278 (0.850 - 1.923), 0.2379 | 1.158 (0.782 - 1.715), 0.4636 |
|  |  | ATA | 0.066 | 0.080, 0.062 | 1.569 | 0.210 | 0.733 | 1.466 (0.937 - 2.295), 0.0924 | 1.338 (0.868 - 2.063), 0.1861 |
|  |  | GTG | 0.034 | 0.048, 0.030 | 2.682 | 0.102 | 0.407 | 1.780 (1.000 - 3.169), 0.0471 | 1.622 (0.922 - 2.852), 0.0903 |
|  |  | ATG | 0.021 | 0.028, 0.019 | 1.096 | 0.295 | 0.868 | 1.598 (0.756 - 3.376), 0.2155 | 1.440 (0.688 - 3.015), 0.3305 |
|  |  | GCA | 0.012 | 0.021, 0.010 | 3.490 | 0.062 | 0.253 | 2.556 (1.043 - 6.265), 0.0337 | 2.314 (0.952 - 5.626), 0.0570 |

| ^a^ p value calculated using permutation test and a total of 1,000 permutations. |
| --- |
| ^b^ the most common haplotype was used as the reference. |
| ^c^ all other haplotypes pooled together were used as the reference. |

## **Additional file 1: Table S28. Associations between LXRA polymorphic variants and dyslipidemia diagnosed by K/DOQI recommendations^2^ in hemodialysis patients.**

| Genotypes and MAF | Dyslipidemic patients | Non-dyslipidemic patients by K/DOQI | Odds ratio (95% CI) | P value^a^ | P*_trend_*^b^ | P*_genotype_*^a^ |
| --- | --- | --- | --- | --- | --- | --- |
|  | n (% of all) | n (% of all) |  |  |  |  |
| *LXRA* rs2279238, n = 861, P for HWE = 0.920 | | | | | | |
| GG | 304 (66.7) | 291 (71.9) | Reference | - | 0.098 | 0.248 |
| AG | 137 (30.0) | 104 (25.7) | 1.261 (0.933 - 1.704) | 0.131 |  |  |
| AA | 15 (3.3) | 10 (2.5) | 1.436 (0.635 - 3.248) | 0.383 |  |  |
| AA vs GG + AG | | | 1.344 (0.597 - 3.025) | 0.474 |  |  |
| AG + AA vs GG | | | 1.276 (0.954 - 1.708) | 0.100 |  |  |
| MAF | 167 (18.3) | 124 (15.3) | 1.240 (0.962 - 1.599) | 0.097 |  |  |
| P for HWE | 0.928 | 0.845 |  |  |  |  |
| *LXRA* rs7120118, n = 860, P for HWE = 0.447 | | | | | | |
| TT | 205 (45.1) | 204 (50.4) | Reference | - | 0.107 | 0.266 |
| CT | 206 (45.3) | 169 (41.7) | 1.213 (0.916 - 1.607) | 0.178 |  |  |
| CC | 44 (9.7) | 32 (7.9) | 1.368 (0.834 - 2.244) | 0.213 |  |  |
| CC vs TT + CT | | | 1.248 (0.775 -2.009) | 0.362 |  |  |
| CT + CC vs TT | | | 1.238 (0.946 - 1.619) | 0.119 |  |  |
| MAF | 294 (32.3) | 233 (28.8) | 1.182 (0.962 -1.452) | 0.112 |  |  |
| P for HWE | 0.454 | 0.714 |  |  |  |  |
| *LXRA* rs11039155, n = 862, P for HWE = 0.635 | | | | | | |
| GG | 309 (67.8) | 295 (72.8) | Reference | - | 0.132 | 0.265 |
| AG | 133 (29.2) | 99 (24.4) | 1.283 (0.945 - 1.740) | 0.110 |  |  |
| AA | 14 (3.1) | 11 (2.7) | 1.215 (0.543 - 2.719) | 0.635 |  |  |
| AA vs GG + AG | | | 1.135 (0.509 - 2.528) | 0.757 |  |  |
| AG + AA vs GG | | | 1.276 (0.951 - 1.712) | 0.104 |  |  |
| MAF | 161 (17.7) | 121 (14.9) | 1.221 (0.944 - 1.579) | 0.129 |  |  |
| P for HWE | 0.946 | 0.443 |  |  |  |  |

a - Chi^2^ test; b – Chi^2^ Test for Trend in Proportions

## **Additional file 1: Table S29. Associations between LXRA polymorphic variants and atherogenic dyslipidemia in hemodialysis patients.**

| Genotypes and MAF | Atherogenic dyslipidemia | Without atherogenic dyslipidemia | Odds ratio (95% CI) | P value^a^ | P*_trend_*^b^ | P*_genotype_*^a^ |
| --- | --- | --- | --- | --- | --- | --- |
|  | n (% of all) | n (% of all) |  |  |  |  |
| *LXRA* rs2279238, n = 861, P for HWE = 0.920 | | | | | | |
| GG | 290 (65.0) | 305 (73.5) | Reference | - | 0.009^c^ | 0.027^c^ |
| AG | 141 (31.6) | 100 (24.1) | 1.483 (1.096 - 2.007) | 0.010^c^ |  |  |
| AA | 15 (3.4) | 10 (2.4) | 1.578 (0.698 - 3.568) | 0.270 |  |  |
| AA vs GG + AG | | | 1.410 (0.626 - 3.173) | 0.405 |  |  |
| AG + AA vs GG | | | 1.492 (1.114 -1.998) | 0.007^c^ |  |  |
| MAF | 171 (19.2) | 120 (14.5) | 1.403 (1.087 - 1.812) | 0.009^c^ |  |  |
| P for HWE | 0.671 | 0.599 |  |  |  |  |
| *LXRA* rs7120118, n = 860, P for HWE = 0.447 | | | | | | |
| TT | 205 (45.9) | 204 (49.4) | Reference | - | 0.088 | 0.112 |
| CT | 194 (43.4) | 181 (43.8) | 1.067 (0.806 - 1.412) | 0.652 |  |  |
| CC | 48 (10.7) | 28 (6.8) | 1.706 (1.030 - 2.826) | 0.037^c^ |  |  |
| CC vs TT + CT | | | 1.654 (1.017 - 2.691) | 0.041^c^ |  |  |
| CT + CC vs TT | | | 1.152 (0.881 - 1.506) | 0.300 |  |  |
| MAF | 290 (32.4) | 237 (28.7) | 1.193 (0.971 -1.466) | 0.092 |  |  |
| P for HWE | 0.835 | 0.149 |  |  |  |  |
| *LXRA* rs11039155, n = 862, P for HWE = 0.635 | | | | | | |
| GG | 296 (66.4) | 308 (74.2) | Reference | - | 0.010^c^ | 0.035^c^ |
| AG | 134 (30.0) | 98 (23.6) | 1.423 (1.048 - 1.931) | 0.023^c^ |  |  |
| AA | 16 (3.6) | 9 (2.2) | 1.850 (0.805 - 4.251) | 0.142 |  |  |
| AA vs GG + AG | | | 1.679 (0.734 - 3.841) | 0.215 |  |  |
| AG + AA vs GG | | | 1.459 (1.086 - 1.959) | 0.012^c^ |  |  |
| MAF | 166 (18.6) | 116 (14.0) | 1.407 (1.087 - 1.823) | 0.009^c^ |  |  |
| P for HWE | 0.863 | 0.715 |  |  |  |  |

a - Chi^2^ test; b – Chi^2^ Test for Trend in Proportions c – not significant after Bonferroni correction (for 3 SNPs, 7 analyzes for the association in each SNP, and 1 phenotype, a significant Bonferroni corrected P-value is below 0.002)

## **Additional file 1: Table S30. Gene-gene interactions between tested ENHO, RXRA, and *LXRA* polymorphisms in HD patients stratified by dyslipidemia by K/DOQI criteria or atherogenic dyslipidemia.**

| Genes and rs numbers | Testing Balanced Accuracy | | | Cross Validation Consistency | | p value^a^ |  |
| --- | --- | --- | --- | --- | --- | --- | --- |
| **dyslipidemia by K/DOQI criteria = CASES, without dyslipidemia by K/DOQI criteria = CONTROLS** | | | | | | |  |
| *ENHO*_rs2281997, *LXRA*_rs7120118 | 0.5467 | | | 8 / 10 | | 0.171 |  |
| *RXRA*_rs10776909, *ENHO*_rs2281997, *LXRA*_rs7120118 | 0.5641 | | | 9 / 10 | | 0.033* |  |
| *RXRA*_rs10881578, *RXRA*_rs749759, *ENHO*_rs2281997, *LXRA*_rs2279238 | 0.5131 | | | 2 / 10 | | 0.698 |  |
| **atherogenic dyslipidemia = CASES, without atherogenic dyslipidemia = CONTROLS** | | | | | | |  |
|  |  | | |  | |  |  |
| *RXRA*_rs10881578, *LXRA*_rs2279238 | | 0.5631 | 10 / 10 | | 0.036* | | |
| *RXRA*_rs10881578, *ENHO*_rs72735260, *LXRA*_rs2279238 | | 0.5284 | 7 / 10 | | 0.414 | | |
| *RXRA_*rs10881578, *ENHO*_rs72735260, *ENHO*_rs2281997, *LXRA*_rs2279238 | | 0.5020 | 5 / 10 | | 0.804 | | |

^a^ Significance of accuracy, empirical p value based on 1,000 permutations

Significant P-values are indicated using an asterisk.

## **Additional file 1: Table S31. Gene-gene interactions between tested SNPs in respect of myocardial infarction occurrence.**

| **myocardial infarction = CASES** |  |  |  |
| --- | --- | --- | --- |
| **without myocardial infarction = CONTROLS** |  |  |  |
|  |  |  |  |
| **Genes and rs numbers** | **Testing Balanced Accuracy** | **Cross Validation Consistency** | **p value^a^** |
| *RXRA*_rs10776909, *ENHO*_rs2281997 | 0.503 | 6 / 10 | 0.807 |
| *RXRA*_rs10776909, *ENHO*_rs2281997, *LXRA*_rs7120118 | 0.497 | 4 / 10 | 0.862 |
| *RXRA*_rs10881578, *RXRA*_rs749759, *ENHO*_rs2281997, *LXRA*_rs7120118 | 0.508 | 6 / 10 | 0.759 |
|  |  |  |  |
| ^a^ Significance of accuracy, empirical p value based on 1,000 permutations |  |  |  |

## **Additional file 1: Table S32. Results of the scanning of rs10881578, rs749759, rs10776909 and rs11039155 flanking sequences by FIMO software for DNA-binding sites of ENCODE ChIP-seq predicted transcription factors peaks. Table contains only statistically significant DNA-binding sites.**

| **SNP** | **Transcription factor** | **Strand** | **Start** | **End** | **p-value** | **q-value** | **Matched sequence** |
| --- | --- | --- | --- | --- | --- | --- | --- |
| rs10881578 | MYC | "+" | 154 | 163 | 5.42e-05 | 0.0196 | CCACATGCCG |
| rs10881578 | MAX | "+" | 152 | 161 | 6.23e-05 | 0.032 | AGCCACATGC |
| rs749759 | CTCF | "+" | 48 | 68 | 1.48e-06 | 0.000361 | ACGCCCACCAGCAGGCACCCC |
| rs749759 | MYC | "-" | 69 | 78 | 5.1e-05 | 0.0191 | TCACGTGCCG |
| rs10776909 | CTCF | "+" | 189 | 209 | 1.15e-06 | 0.00027 | CTGCCCAGGAGGGGGCTGGAG |
| rs10776909 | EBF1 | "+" | 191 | 201 | 2.62e-05 | 0.0109 | GCCCAGGAGGG |
| rs10776909 | Elf-1 | "-" | 250 | 262 | 4.44e-05 | 0.00953 | GTGGCAGGAAATG |
| rs10776909 | RELA | "+" | 310 | 323 | 1.53e-06 | 0.00199 | TGGGGGTTCCCCAG |
| rs10776909 | RELA | "-" | 309 | 322 | 7.06e-05 | 0.0305 | TGGGGAACCCCCAC |
| rs11039155 | MAZ | "-" | 562 | 578 | 3.32e-06 | 0.00587 | TCAGGGGAGAGGGATGG |
| rs11039155 | MAZ | "-" | 737 | 753 | 2.87e-05 | 0.0194 | GGGGAGGACTGGGCGGG |
| rs11039155 | MAZ | "-" | 567 | 583 | 3.3e-05 | 0.0194 | TGGGCTCAGGGGAGAGG |
| rs11039155 | MAZ | "-" | 632 | 648 | 3.89e-05 | 0.0225 | GGAGGAAGGAAGAAGGC |
| rs11039155 | MAZ | "-" | 742 | 758 | 5.29e-05 | 0.0234 | AGACTGGGGAGGACTGG |
| rs11039155 | MAZ | "+" | 403 | 419 | 7.19e-05 | 0.025 | GGAGAAGGGAGCTGAGG |
| rs11039155 | CTCF | "+" | 640 | 660 | 2.12e-05 | 0.0367 | CCTTCCTCCAGAGAGCAGTCC |
| rs11039155 | CTCF | "+" | 641 | 659 | 2.56e-05 | 0.0347 | CTTCCTCCAGAGAGCAGTC |
| rs11039155 | CTCF | "-" | 972 | 990 | 3.54e-05 | 0.0315 | CCACCCTTAGAGGGCAGCA |

## **Additional file 1: Table S33. Function annotation information for rs749759, rs72735260, rs10881578, rs10776909, rs2281997, rs2279238, rs7120118, and rs11039155 SNPs (annotation comes from dbSNP's predicted functional effect of variant on RefSeq transcripts).**

| **SNP** | **ChIP-seq overlapping** |
| --- | --- |
| rs749759 | both MYC and CTCF lie upstream of this SNP |
| rs72735260 | lies in DHS1 cluster, present in Th1 cells |
| rs10881578 | Overlaps MAX::MYC transfacs, but we don't have them in our dataset to test, also in DHS1 cluster expressed in 19 cell lines, as well as overlaps histone modification site H3K27Ac in 9 cell lines |
| rs10776909 | Overlaps multiple transfacs (POLR2A, CTCF, RELA, Elf-1, EBF1) and DHS1 present in 41 out of 125 cell lines, histone modification H3K27Ac in 7 cell lines |
| rs2281997 | overlaps DHS1 site expressed in Th1 cells |
| rs2279238* | No overlapping ChIP-seq ENCODE signals nor DHs clusters |
| rs7120118 | Overlaps DHS cluster expressed in multiple cell lines and ENCDOE ChIP-seq signal for TAL-1 |
| rs11039155 | Overlaps DHs cluster and multiple ENCODE TF ChIP-seq signals (MAZ, CTCF, SMC-3) and close proximity with MAX, RAD21, POLR2A, RCOR1) |

*rs2279238 is placed on the reverse strand

## **Additional file 1: Table S34. FIMO predicted DNA-binding sites overlapping variants positions.**

| **Database** | **SNP/allele** | **Transcription**  **factor** | **Motif name** | **Start** | **End** |  | **Strand** | **Score** | **p-value** | **q-value** | **Matched sequence** |
| --- | --- | --- | --- | --- | --- | --- | --- | --- | --- | --- | --- |
| CIS-BP | rs749759_G | NR0B1 | M6381_1.02 | 146 | 155 |  | "-" | 11.2321 | 1.96e-05 | 0.022 | CCTCCCACTC |
| CIS-BP | rs749759_G | Sp4 | M6483_1.02 | 136 | 159 |  | "+" | 12.1381 | 2.54e-05 | 0.0266 | GGGGCCAGGGGAGTGgGAGGCACG |
| CIS-BP | rs749759_G | EGR-1 | M4459_1.02 | 144 | 163 |  | "+" | 11.2849 | 5.48e-05 | 0.0587 | GGGAGTGgGAGGCACGGAGG |
| CIS-BP | rs749759_G | ZBTB7B | M6539_1.02 | 136 | 157 |  | "+" | 10.9721 | 6.36e-05 | 0.0226 | GGGGCCAGGGGAGTGgGAGGCA |
| CIS-BP | rs749759_G | PLAG1 | M6420_1.02 | 151 | 167 |  | "+" | 10.8198 | 7.38e-05 | 0.078 | gGAGGCACGGAGGTGGG |
| CIS-BP | rs749759_G | EGR-2 | M6199_1.02 | 145 | 155 |  | "+" | 10.8 | 8.97e-05 | 0.033 | GGAGTGgGAGG |
| HOCOMOCOv9 | rs749759_G | NR0B1 | NR0B1_si | 146 | 155 |  | "+" | 12.0273 | 1.93e-05 | 0.0217 | GAGTGgGAGG |
| HOCOMOCOv9 | rs749759_G | PLAG1 | PLAG1_f1 | 151 | 167 |  | "+" | 11.1683 | 6.95e-05 | 0.0513 | gGAGGCACGGAGGTGGG |
| HOCOMOCOv9 | rs749759_A | PLAG1 | PLAG1_f1 | 151 | 167 |  | "+" | 10.6337 | 9.66e-05 | 0.0513 | aGAGGCACGGAGGTGGG |
| JASPAR | rs749759_G | EGR-1 | MA0162.2 | 145 | 158 |  | "-" | 11.6038 | 6.36e-05 | 0.0698 | GTGCCTCCCACTCC |
| JASPAR | rs749759_G | Sp3 | MA0746.1 | 142 | 152 |  | "-" | 11.056 | 7.23e-05 | 0.0255 | CCCACTCCCCT |
| JASPAR | rs749759_G | EGR-2 | MA0472.1 | 144 | 158 |  | "-" | 11.2295 | 7.52e-05 | 0.0835 | GTGCCTCCCACTCCC |
| JASPAR | rs749759_G | EWSR1-FLI1 | MA0149.1 | 150 | 167 |  | "+" | -14.5405 | 9.17e-05 | 0.0987 | GgGAGGCACGGAGGTGGG |
|  |  |  |  |  |  |  |  |  |  |  |  |
| CIS-BP | rs72735260_T | NR3C1 | M4428_1.02 | 141 | 154 |  | "-" | 10.8902 | 6.45e-05 | 0.0722 | GGTACACTCTTTCC |
| CIS-BP | rs72735260_T | AR | M1841_1.02 | 139 | 153 |  | "-" | 10.3665 | 9.9e-05 | 0.111 | GTACACTCTTTCCCT |
| HOCOMOCOv9 | rs72735260 | - | - | - | - |  | - | - | - | - | - |
| JASPAR | rs72735260_T | AR | MA0007.3 | 139 | 155 |  | "+" | 9.7193 | 2.41e-05 | 0.0273 | AGGGAAAGAGTGtACCC |
| JASPAR | rs72735260_T | RARA::RXRA | MA0159.1 | 151 | 167 |  | "-" | 11.0103 | 3.85e-05 | 0.0423 | GGGTCAGGGGCCGGGTA |
| JASPAR | rs72735260_T | NR3C1 | MA0113.3 | 139 | 155 |  | "-" | 4.7551 | 6.65e-05 | 0.0733 | GGGTACACTCTTTCCCT |
|  |  |  |  |  |  |  |  |  |  |  |  |
| CIS-BP | rs10881578_A | ENO1 | M6210_1.02 | 151 | 163 |  | "+" | 10.325 | 9.19e-05 | 0.103 | aAGCCACATGCCG |
| HOCOMOCOv9 | rs10881578_A | TLX1 | TLX1_f2 | 148 | 164 |  | "-" | 10.6742 | 7.12e-05 | 0.0778 | ACGGCATGTGGCTTAGG |
| HOCOMOCOv9 | rs10881578_A | ENOA | ENOA_si | 151 | 163 |  | "-" | 10.9583 | 8.48e-05 | 0.0935 | CGGCATGTGGCTT |
| JASPAR | rs10881578_A | Crx | MA0467.1 | 149 | 159 |  | "-" | 10.5088 | 7.76e-05 | 0.087 | ATGTGGCTTAG |
|  |  |  |  |  |  |  |  |  |  |  |  |
| CIS-BP | rs10776909_T | RREB1 | M6456_1.02 | 138 | 159 |  | "+" | 14.443 | 4.93e-06 | 0.00521 | AGGGATGTGGGGAtTTTGAGTT |
| CIS-BP | rs10776909_C | RREB1 | M6456_1.02 | 138 | 159 |  | "+" | 12.0506 | 2.45e-05 | 0.0129 | AGGGATGTGGGGAcTTTGAGTT |
| CIS-BP | rs10776909_C | IRF-8 | M6313_1.02 | 149 | 163 |  | "-" | 14.2548 | 5.64e-06 | 0.00351 | GGGGAACTCAAAGTC |
| CIS-BP | rs10776909_T | IRF-8 | M6313_1.02 | 149 | 163 |  | "-" | 14.0828 | 6.43e-06 | 0.00351 | GGGGAACTCAAAATC |
| CIS-BP | rs10776909_T | ETS1 | M4461_1.02 | 140 | 160 |  | "+" | 12.8654 | 6.67e-06 | 0.00413 | GGATGTGGGGAtTTTGAGTTC |
| CIS-BP | rs10776909_C | ETS1 | M4461_1.02 | 140 | 160 |  | "+" | 12.6346 | 7.54e-06 | 0.00413 | GGATGTGGGGAcTTTGAGTTC |
| CIS-BP | rs10776909_T | SMARCC2 | M4527_1.02 | 140 | 160 |  | "-" | 12.0962 | 9.95e-06 | 0.00567 | GAACTCAAAATCCCCACATCC |
| CIS-BP | rs10776909_C | SMARCC2 | M4527_1.02 | 140 | 160 |  | "-" | 12.0321 | 1.03e-05 | 0.00567 | GAACTCAAAGTCCCCACATCC |
| CIS-BP | rs10776909_T | BCL11A | M4453_1.02 | 151 | 165 |  | "-" | 13.8526 | 7.66e-06 | 0.00706 | GAGGGGAACTCAAAA |
| CIS-BP | rs10776909_C | BCL11A | M4453_1.02 | 151 | 165 |  | "-" | 13.109 | 1.31e-05 | 0.00706 | GAGGGGAACTCAAAG |
| CIS-BP | rs10776909_C | NR3C1 | M4428_1.02 | 147 | 160 |  | "+" | 13.1519 | 1.16e-05 | 0.013 | GGGAcTTTGAGTTC |
| CIS-BP | rs10776909_C | AR | M1841_1.02 | 148 | 162 |  | "+" | 11.6218 | 4,00E-05 | 0.0448 | GGAcTTTGAGTTCCC |
| CIS-BP | rs10776909_T | NR2E3 | M6391_1.02 | 147 | 160 |  | "+" | 11.0764 | 4.52e-05 | 0.05 | GGGAtTTTGAGTTC |
| CIS-BP | rs10776909_C | IRF-5 | M6311_1.02 | 148 | 167 |  | "-" | 6.92949 | 4.59e-05 | 0.0246 | GAGAGGGGAACTCAAAGTCC |
| CIS-BP | rs10776909_C | HNF-4-gamma | M2286_1.02 | 147 | 161 |  | "+" | 11.1154 | 5.49e-05 | 0.06 | GGGAcTTTGAGTTCC |
| CIS-BP | rs10776909_T | MZF-1 | M6356_1.02 | 143 | 151 |  | "+" | 11.0573 | 6.33e-05 | 0.023 | TGTGGGGAt |
| CIS-BP | rs10776909_C | IRF-1 | M1882_1.02 | 146 | 166 |  | "-" | 9.0641 | 7.7e-05 | 0.0835 | AGAGGGGAACTCAAAGTCCCC |
| CIS-BP | rs10776909_T | LCOR (*Meleagris_gallopavo*) | M1507_1.02 | 150 | 159 |  | "+" | 8.86164 | 8.72e-05 | 0.0994 | AtTTTGAGTT |
| CIS-BP | rs10776909_C | HNF-4-alpha | M4698_1.02 | 146 | 160 |  | "+" | 10.3228 | 9.28e-05 | 0.103 | GGGGAcTTTGAGTTC |
| HOCOMOCOv9 | rs10776909_T | RREB1 | RREB1_si | 138 | 159 |  | "+" | 15.1264 | 3.19e-06 | 0.00346 | AGGGATGTGGGGAtTTTGAGTT |
| HOCOMOCOv9 | rs10776909_C | RREB1 | RREB1_si | 138 | 159 |  | "+" | 12.6667 | 1.29e-05 | 0.00699 | AGGGATGTGGGGAcTTTGAGTT |
| HOCOMOCOv9 | rs10776909_C | IRF-8 | IRF-8_si | 149 | 163 |  | "-" | 14.2529 | 6.54e-06 | 0.00413 | GGGGAACTCAAAGTC |
| HOCOMOCOv9 | rs10776909_T | IRF-8 | IRF-8_si | 149 | 163 |  | "-" | 14.023 | 7.54e-06 | 0.00413 | GGGGAACTCAAAATC |
| HOCOMOCOv9 | rs10776909_C | IRF-1 | IRF-1_si | 150 | 161 |  | "-" | 11.1081 | 2.61e-05 | 0.0239 | GGAACTCAAAGT |
| HOCOMOCOv9 | rs10776909_T | IRF-1 | IRF-1_si | 150 | 161 |  | "-" | 9.83784 | 4.23e-05 | 0.0239 | GGAACTCAAAAT |
| HOCOMOCOv9 | rs10776909_T | NR2E3 | NR2E3_f1 | 147 | 160 |  | "-" | 11.3053 | 3.34e-05 | 0.0364 | GAACTCAAAATCCC |
| HOCOMOCOv9 | rs10776909_C | IRF-5 | IRF-5_f1 | 148 | 167 |  | "-" | -0.760684 | 4.69e-05 | 0.0508 | GAGAGGGGAACTCAAAGTCC |
| HOCOMOCOv9 | rs10776909_T | MZF-1 | MZF-1_f1 | 143 | 151 |  | "+" | 11.675 | 6.67e-05 | 0.0244 | TGTGGGGAt |
| HOCOMOCOv9 | rs10776909_C | GCR | GCR_do | 145 | 162 |  | "-" | 10.3582 | 9.75e-05 | 0.106 | GGGAACTCAAAGTCCCCA |
| JASPAR | rs10776909_C | NR3C1 | MA0113.2 | 147 | 161 |  | "-" | 15.0615 | 4.37e-06 | 0.00479 | GGAACTCAAAGTCCC |
| JASPAR | rs10776909_C | NR3C2 | MA0727.1 | 146 | 162 |  | "-" | 8.65217 | 1.72e-05 | 0.0161 | GGGAACTCAAAGTCCCC |
| JASPAR | rs10776909_C | NR3C2 | MA0727.1 | 146 | 162 |  | "+" | 7.02174 | 2.82e-05 | 0.0161 | GGGGAcTTTGAGTTCCC |
| JASPAR | rs10776909_C | AR | MA0007.2 | 148 | 162 |  | "-" | 12.1176 | 2.96e-05 | 0.0333 | GGGAACTCAAAGTCC |
| JASPAR | rs10776909_C | HNF-4-alpha | MA0114.2 | 146 | 160 |  | "+" | 11.2917 | 4.13e-05 | 0.0455 | GGGGAcTTTGAGTTC |
| JASPAR | rs10776909_C | AR | MA0007.1 | 143 | 164 |  | "-" | 10.4062 | 4.24e-05 | 0.0473 | AGGGGAACTCAAAGTCCCCACA |
| JASPAR | rs10776909_C | HNF-4-gamma | MA0484.1 | 147 | 161 |  | "-" | 11.1569 | 4.6e-05 | 0.0498 | GGAACTCAAAGTCCC |
| JASPAR | rs10776909_C | NR3C1 | MA0113.3 | 146 | 162 |  | "-" | 5.125 | 4.91e-05 | 0.034 | GGGAACTCAAAGTCCCC |
| JASPAR | rs10776909_C | IRF-1 | MA0050.1 | 150 | 161 |  | "-" | 8.46316 | 4.96e-05 | 0.0498 | GGAACTCAAAGT |
| JASPAR | rs10776909_T | IRF-1 | MA0050.1 | 150 | 161 |  | "-" | 6.78947 | 8.76e-05 | 0.0498 | GGAACTCAAAAT |
| JASPAR | rs10776909_C | STAT1 | MA0137.1 | 149 | 162 |  | "-" | 6.21053 | 5.6e-05 | 0.0628 | GGGAACTCAAAGTC |
| JASPAR | rs10776909_C | NR3C1 | MA0113.3 | 146 | 162 |  | "+" | 4.4375 | 5.98e-05 | 0.034 | GGGGAcTTTGAGTTCCC |
| JASPAR | rs10776909_C | AR | MA0007.3 | 146 | 162 |  | "+" | 6.03509 | 7.38e-05 | 0.0523 | GGGGAcTTTGAGTTCCC |
| JASPAR | rs10776909_T | Dmbx1 | MA0883.1 | 142 | 158 |  | "+" | 9.1519 | 9,00E-05 | 0.102 | ATGTGGGGAtTTTGAGT |
| JASPAR | rs10776909_C | AR | MA0007.3 | 146 | 162 |  | "-" | 5.21053 | 9.55e-05 | 0.0523 | GGGAACTCAAAGTCCCC |
|  |  |  |  |  |  |  |  |  |  |  |  |
| CIS-BP | rs2281997_T | HOXD13 | M6302_1.02 | 149 | 159 |  | "+" | 10.3902 | 8.23e-05 | 0.0956 | TAtACAGTAAA |
| HOCOMOCOv9 | rs2281997_T | HXD13 | HXD13_f1 | 149 | 159 |  | "-" | 10.7653 | 8.14e-05 | 0.0936 | TTTACTGTATA |
| JASPAR | rs2281997_T | Tcf7 | MA0769.1 | 150 | 161 |  | "+" | 7.40984 | 7.92e-05 | 0.0884 | AtACAGTAAAGG |
| JASPAR | rs2281997_C | HNF-1-beta | MA0153.1 | 147 | 158 |  | "-" | 6.62617 | 9.86e-05 | 0.114 | TTACTGTGTACC |
|  |  |  |  |  |  |  |  |  |  |  |  |
| CIS-BP | rs2279238_A | ZBTB3 *(Mus musculus*) | M0442_1.02 | 144 | 152 |  | "+" | 10.1852 | 8.54e-06 | 0.00974 | TATGCAGtG |
| CIS-BP | rs2279238_A | ZSCAN4 | M5981_1.02 | 144 | 158 |  | "+" | 12.5988 | 1.3e-05 | 0.0147 | TATGCAGtGTGTGTG |
| CIS-BP | rs2279238_A | Klf8 | M6326_1.0 | 148 | 156 |  | "+" | 12.6566 | 2.29e-05 | 0.0263 | CAGtGTGTG |
| HOCOMOCOv9 | rs2279238_A | Klf8 | Klf8_f1 | 148 | 156 |  | "+" | 13.119 | 2.29e-05 | 0.0263 | CAGtGTGTG |
| JASPAR | rs2279238_A | EGR-2 | MA0472.1 | 148 | 162 |  | "-" | 12.0484 | 4.41e-05 | 0.0273 | TCCCCACACACACTG |
| JASPAR | rs2279238_G | EGR-2 | MA0472.1 | 148 | 162 |  | "-" | 11.9032 | 4.85e-05 | 0.0273 | TCCCCACACACGCTG |
|  |  |  |  |  |  |  |  |  |  |  |  |
| CIS-BP | rs7120118_C | IRF-4 | M4463_1.02 | 145 | 162 |  | "-" | 12.7879 | 7.59e-06 | 0.00841 | ACTCATGAAATGAGAAAT |
| HOCOMOCOv9 | rs7120118_T | IRF-4 | IRF4_si | 141 | 156 |  | "-" | 10.3729 | 9.09e-05 | 0.1 | GAAATAAGAAATGCAA |
| JASPAR | rs7120118 | - | - | - | - |  | - | - | - | - | - |
|  |  |  |  |  |  |  |  |  |  |  |  |
| CIS-BP | rs11039155_G | ETV7 | M6224_1.02 | 144 | 160 |  | "+" | 11.2695 | 4.19e-05 | 0.0459 | GCTCCAGgAAGAGATGT |
| CIS-BP | rs11039155_G | Elf-1 | M2275_1.02 | 144 | 156 |  | "+" | 11.5569 | 4.59e-05 | 0.0515 | GCTCCAGgAAGAG |
| HOCOMOCOv9 | rs11039155_G | ETV7 | ETV7_si | 144 | 160 |  | "+" | 8.66364 | 6.3e-05 | 0.0695 | GCTCCAGgAAGAGATGT |
| HOCOMOCOv9 | rs11039155_G | Elf-1 | Elf-1_f1 | 147 | 156 |  | "+" | 11.378 | 7.73e-05 | 0.0865 | CCAGgAAGAG |
| HOCOMOCOv9 | rs11039155_G | GABP1+GABP2 | GABP1+GABP2_f1 | 148 | 157 |  | "+" | 11.1444 | 9.71e-05 | 0.108 | CAGgAAGAGA |
| JASPAR | rs11039155_G | Stat3 | MA0144.1 | 145 | 154 |  | "+" | 12.3636 | 2.64e-05 | 0.0293 | CTCCAGgAAG |
| JASPAR | rs11039155_G | NR3C1 | MA0113.1 | 147 | 164 |  | "-" | 11.5179 | 4.11e-05 | 0.0239 | AAGGACATCTCTTCCTGG |
| JASPAR | rs11039155_G | Elf-1 | MA0473.1 | 144 | 156 |  | "+" | 12.0204 | 4.34e-05 | 0.0484 | GCTCCAGgAAGAG |
| JASPAR | rs11039155_A | NR3C1 | MA0113.1 | 147 | 164 |  | "-" | 11.2768 | 4.67e-05 | 0.0239 | AAGGACATCTCTTTCTGG |
| JASPAR | rs11039155_G | GABPA | MA0062.1 | 147 | 156 |  | "+" | 11.6364 | 6.61e-05 | 0.0738 | CCAGgAAGAG |

## **Additional file 1: Table S35. Gene-gene interactions between *ENHO* and T helper 1 cell cytokine gene polymorphisms by MDR analysis.**

| HD patients without dyslipidemia by K/DOQI = CONTROLS |  |  |  |
| --- | --- | --- | --- |
| HD patients with dyslipidemia by K/DOQI = CASES |  |  |  |
|  |  |  |  |
| **Genes and rs numbers** | **Testing Balanced Accuracy** | **Cross Validation Consistency** | **p value^a^** |
| *ENHO*_rs2281997, *IL18*_rs360719 | 0.5765 | 10 / 10 | 0.004* |
| *ENHO*_rs2281997, *IL12A*_rs568408, *IL18*_rs360719 | 0.5296 | 7 / 10 | 0.410 |
| *ENHO*_rs72735260, *ENHO*_rs2281997, *IL12A*_rs568408, *IL18*_rs360719 | 0.5286 | 7 / 10 | 0.427 |
|  |  |  |  |
| ^a^Significance of accuracy, empirical p value based on 1,000 permutations | |  |  |
|  |  |  |  |
| HD patients without atherogenic dyslipidemia = CONTROLS |  |  |  |
| HD patients with atherogenic dyslipidemia = CASES |  |  |  |
|  |  |  |  |
| **Genes and rs numbers** | **Testing Balanced Accuracy** | **Cross Validation Consistency** | **p value^a^** |
| *ENHO*_rs2281997, *IL12A*_rs568408 | 0.5681 | 10 / 10 | 0.013* |
| *ENHO*_rs2281997, *IL12A*_rs568408, *IL18*_rs360719 | 0.5066 | 5 / 10 | 0.743 |
| *ENHO*_rs72735260, *ENHO*_rs2281997, *IL12A*_rs568408, *IL12B*_rs3212227 | 0.5318 | 6 / 10 | 0.348 |
|  |  |  |  |
| ^a^ Significance of accuracy, empirical p value based on 1,000 permutations | |  |  |

Significant P-values are indicated using an asterisk.

# Additional file 1: Supplementary Figures

# Additional file 1: Figure S1 Adropin concentrations according to types of dyslipidemia and *ENHO* rs2281997 genotypes.

# Not adjusted P-values and those adjusted for body mass index are shown.

#
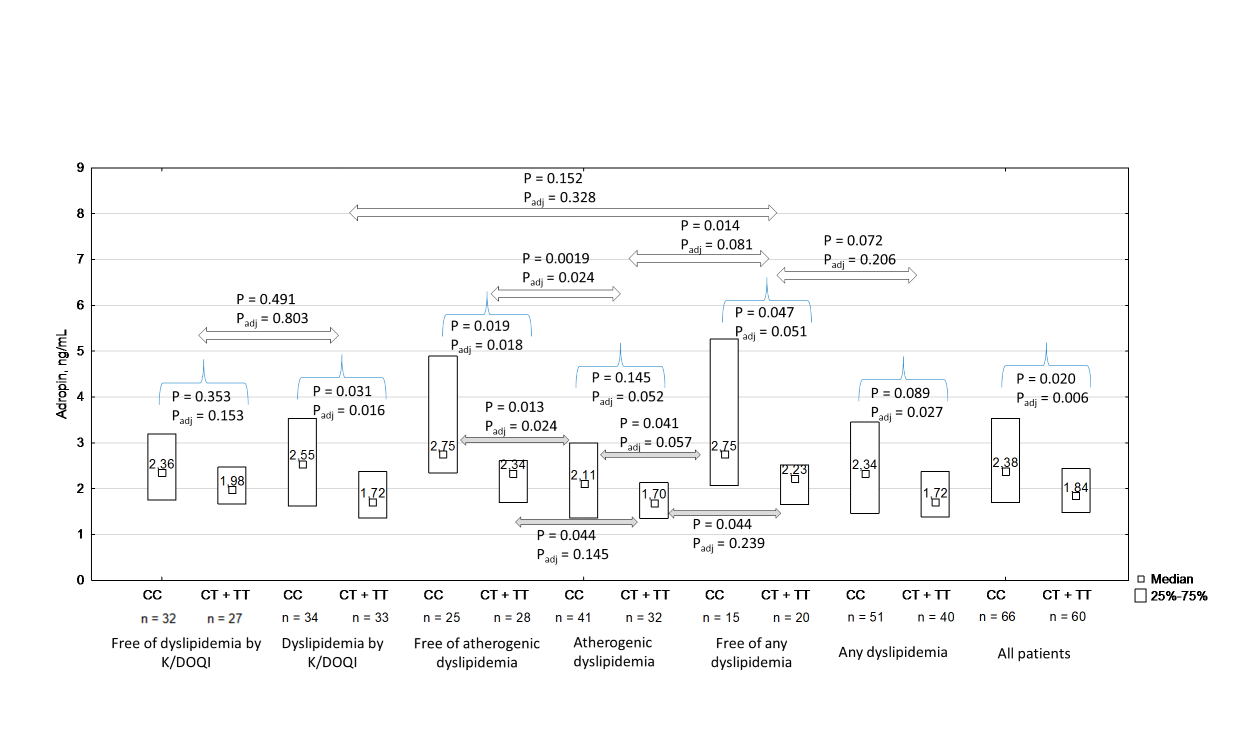


#
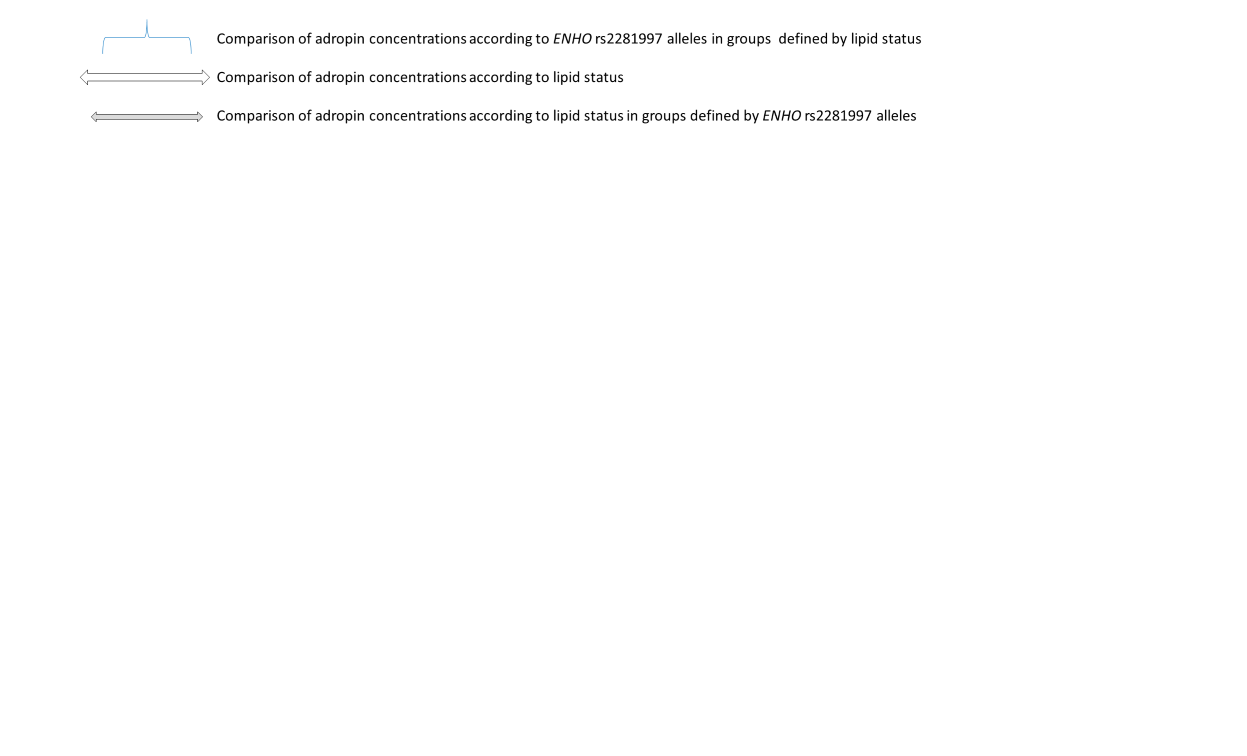


## **Additional file 1:** Figure S2**. Survival probability in HD patients in respect of *IL12A* rs568408 polymorphic variants (the dominant model of inheritance)**

Survival probability was analyzed using the Kaplan-Meier method in 440 patients prospectively followed through 7.5 years. In the Cox analysis, HR was 1.32, 95% CI 1.03 - 1.69, P = 0.028.
